# Gold nanoparticles exhibit anti-osteoarthritic effects via modulating interaction of the “microbiota-gut-joint” axis

**DOI:** 10.1186/s12951-024-02447-y

**Published:** 2024-04-08

**Authors:** Zihan Deng, Chuan Yang, Tingwen Xiang, Ce Dou, Dong Sun, Qijie Dai, Zhiguo Ling, Jianzhong Xu, Fei Luo, Yueqi Chen

**Affiliations:** 1 Department of Orthopedics, Southwest Hospital, Third Military Medical University (Army Medical University), Chongqing, People’s Republic of China; 2 Department of Biomedical Materials Science, Third Military Medical University (Army Medical University), Chongqing, People’s Republic of China; 3 Institute of Immunology, Third Military Medical University (Army Medical University), Chongqing, People’s Republic of China; 4 Department of Orthopedics, Chinese PLA 76th Army Corps Hospital, Xining, People’s Republic of China

**Keywords:** Osteoarthritis (OA), Gold nanoparticles (GNPs), Gut microbiota, Short-chain fatty acid (SCFA) metabolism, Anti-inflammatory effects

## Abstract

Osteoarthritis (OA) is a common degenerative joint disease that can cause severe pain, motor dysfunction, and even disability. A growing body of research indicates that gut microbiota and their associated metabolites are key players in maintaining bone health and in the progression of OA. Short-chain fatty acids (SCFAs) are a series of active metabolites that widely participate in bone homeostasis. Gold nanoparticles (GNPs) with outstanding anti-bacterial and anti-inflammatory properties, have been demonstrated to ameliorate excessive bone loss during the progression of osteoporosis (OP) and rheumatoid arthritis (RA). However, the protective effects of GNPs on OA progression are not clear. Here, we observed that GNPs significantly alleviated anterior cruciate ligament transection (ACLT)-induced OA in a gut microbiota-dependent manner. 16S rDNA gene sequencing showed that GNPs changed gut microbial diversity and structure, which manifested as an increase in the abundance of *Akkermansia* and *Lactobacillus*. Additionally, GNPs increased levels of SCFAs (such as butyric acid), which could have improved bone destruction by reducing the inflammatory response. Notably, GNPs modulated the dynamic balance of M1/M2 macrophages, and increased the serum levels of anti-inflammatory cytokines such as IL-10. To sum up, our study indicated that GNPs exhibited anti-osteoarthritis effects via modulating the interaction of “microbiota-gut-joint” axis, which might provide promising therapeutic strategies for OA.

**Supplementary Information:**

The online version contains supplementary material available at 10.1186/s12951-024-02447-y.

## Introduction

Osteoarthritis (OA) is the most prevalent chronic joint disease, with a prolonged course and a high disability rate, which may result in severe pain, motor dysfunction, and even disability, causing significant implications for individuals’ quality of life and socioeconomic costs [1]. With the increase in the ageing and obese population, OA has emerged as an important public health problem, which currently plagues an estimated 500 million people worldwide [2]. The development of OA is often complicated by the intervention of various mechanical, inflammatory, and metabolic factors. Generally, the pathogenic mechanism of OA is closely related to the disruption of joint homeostasis, mainly affecting articular and periarticular tissues, including articular cartilage, subchondral bone, and synovium [3]. It is characterized by progressive articular cartilage destruction, frequently along with cartilage matrix degradation, subchondral bone loss, as well as vascular invasion, and sensory nerve implantation [4]. Meanwhile, excessive activation of osteoclasts (OCs) is associated with severe bone erosion, and actively involved in the dynamic modulation of bone homeostasis [3]. Therefore, OA is often considered a heterogeneous disease with a wide range of potential pathogenic pathways. The corresponding treatment strategies for OA are relatively limited, and most of them are symptomatic therapies, including moderate exercise and rehabilitation strategies, pharmacological interventions, and joint replacement surgery in late-stage OA [5]. However, patients with OA tend to show a gradual decline in long-term adherence to individualized exercise therapy as time progresses. The most common pharmacological agents used for OA, including nonsteroidal anti-inflammatory drugs (NSAIDs), selective cyclooxygenase-2 (COX-2) inhibitors, and opioids, have been universally recognized to possess considerable toxicity, especially gastrointestinal and cardiovascular complications, as well as the risk of addiction [1, 4, 6]. Therefore, it is necessary to develop a disease-alleviating therapeutic strategy with fewer side effects. In recent years, the important role of gut microbiota in maintaining host physiological homeostasis has attracted increasing attention, and gut microbiota therapy is considered a promising area that can help improve the limitations of OA treatment [7]. Accumulating evidence suggests that alterations in gut microbiota and its metabolites are associated with the occurrence and development of various bone destruction diseases, including rheumatoid arthritis (RA) and osteoporosis (OP) [8, 9]. Recent studies have also shown that gut microbiota and its metabolites exert a significant effect on regulating bone metabolism and affecting the progression of OA [10]. Surprisingly, multiple population-based studies revealed dramatic alterations in the composition and diversity of the intestinal microbial population in patients with OA, which specifically manifested as an increase in *Bilophila*, *Desulfovibrio*, *Clostridium*, *Streptococcus* and *Clostridium spp*, as well as a decrease in *Roseburia*, *Bifidobacterium longum* and *Faecalibacterium prausnitzii*, resulting in a disruption of the dynamic balance of gut microbiota [10–13]. In addition, as for the research of animal models, on the one hand, the dysbiosis of the intestinal microflora can activate a chronic low-grade pro-inflammatory state, thus leading to the destruction of cartilage structure [14]. Interestingly, on the other hand, antibiotics-induced gut microbiota imbalance has been identified to reduce inflammatory responses and negatively modulate the expression of matrix metalloprotease-13 (MMP-13), thereby alleviating the progression of OA [15]. Several researches have shown that oral supplementation with probiotics can reduce adverse lesions in the cartilage structure and provide a joint protective effect in animal models of OA [16]. Moreover, it has also been demonstrated in humans that maintaining the homeostasis of normal intestinal flora acts as a dramatic factor in preventing the development of OA [17, 18]. Gut microbiota-derived metabolites are the key bridges between gut health and joint disease, affecting the intricate balance of systemic inflammatory responses and immune regulation. These metabolites, particularly short-chain fatty acids (SCFAs), have been proved to influence the integrity of the gut barrier, modulate the activity of various immune cells, and orchestrate the production of anti-inflammatory cytokines, participating the progression of multiple diseases including autoimmune diseases, neuropsychiatric disorders, and metabolic diseases [19–21]. Consequently, gut microbial SCFAs may have direct implications on the pathogenesis of OA, presenting an interesting target for potentially mitigating joint degeneration and relieving the symptoms associated with this condition.

As exquisite byproducts of microbial metabolism, the majority of SCFAs are generated by the fermentation of dietary fiber through intricate glycolysis and pentose phosphate pathways under the orchestration of gut microbiota. The types and amounts of SCFAs primarily rely on the gut microbiota composition, digestion time, metabolic capacity of host microorganisms, and fiber content in food. Multiple microbial groups such as *Lactobacillus*, *Bacteroides*, *Prevotella*, *Akkermansia*, and *Eubacterium* could generate a great deal of SCFAs [22–25]. In general, when dietary changes, gut microbiota disorders, or alterations in intestinal permeability occur, the types and content of SCFAs in the intestine will undergo significant changes. Among them, acetic acid, propionic acid, and butyric acid have been widely confirmed by multiple studies to be related to bone metabolism [26, 27]. Recent evidence has indicated that the levels of SCFAs (especially butyric acid) is negatively correlated with the progression of the articular cartilage matrix destruction in the context of OA [28]. Moreover, inactivated *Lactobacillus* (LA-1) ingestion could contribute to an increase in the abundance of SCFA (butyrate)-producing bacteria *Faecalibacterium*, which increases SCFA (butyric acid) levels and relieves OA-related pain by inhibiting the production of pro-inflammatory cytokines as well as reducing cartilage degradation [29]. Although the underlying pathogenesis of OA has not been entirely elucidated, accumulating research indicates that excessive inflammatory responses and immune disorders act as important factors in the destruction of cartilage and joint pain in OA. Therefore, secreted inflammatory molecules, such as pro-inflammatory cytokines exhibit a dramatic effect in the disturbed metabolism of joint tissue involved in OA. It is reported that in particular, interleukin (IL)-1β, tumor necrosis factor (TNF)-α, and IL-6 are the primary pro-inflammatory cytokines participating in the pathophysiology of OA, but other cytokines, such as IL-15, IL-17, IL-18, IL-21, leukemia inhibitory factor (LIF), and chemokines have also been linked to this [30, 31]. Numerous studies have shown that SCFAs can negatively modulate pro-inflammatory cytokines like TNF-α and IL-6, as well as reduce the production of MMP-13, which reveals the protective effects of SCFAs on the aberrant inflammatory reaction along with the degeneration of articular cartilage matrix [32, 33]. Hence, ameliorating the dysbiosis of gut microbiota and increasing the content of active metabolites (SCFAs) in OA can further alleviate clinical symptoms and retard the progress of this disease. Nevertheless, the potential effects of SCFAs on the modulation of bone homeostasis via the “microbiota-gut-joint” axis and their specific molecular mechanisms remain unclear.

With the development of nanotechnology, nanomaterials have become a promising class of novel biomedical therapeutics for a variety of diseases due to their unique biological properties, such as the regulation of cell-cell crosstalk, mutual mediation of biomolecules, catalytic amplification of biochemical reactions, and transduction of biological signals [34]. Metallic nanoparticles (MNPs), a series of widespread biomedical materials with “self-therapeutic” properties, can easily cross the intestinal barrier, intervene in intestinal homeostasis, and even diffuse to other organs, thereby inducing immune responses [34]. Recent studies have confirmed that many MNPs, such as zinc oxide (ZnO), titanium dioxide (TiO_2_), and silver nanoparticles (SNPs) can aggregate in the gut, modulating metabolome and microbiome [35]. Notably, spherical or quasi-spherical gold nanoparticles (GNPs) have been extensively applied in various fields, including biolabeling and detection, clinical diagnostics and therapeutics, as well as drug delivery and imaging, due to their inherent characteristics, such as stable shape-related optoelectronic properties, excellent bio-compatibility, and low bio-toxicity, as well as high drug-loading density surface area [36]. The interaction between GNPs and gut microbiota is an intricate process encompassing a myriad of molecular and cellular mechanisms. According to metagenomic analysis, the microbial community structure is significantly changed under the intervention of GNPs, specifically with an increase in the abundance of *Proteobacteria*, *Bacteroidetes*, *and Firmicutes*, and a decrease in the abundance of *actinobacteria* [37]. And, the small size and distinctive surface properties of GNPs enable them to penetrate the mucus layer, reaching the intestinal epithelium where they directly interact with microbial cell membranes, impacting the growth and metabolism of specific bacteria within the gut microbiota [38, 39]. Based on in-depth investigations, GNPs have been proved to exhibit inherent anti-bacterial and anti-biofilm properties against *Acinetobacter baumannii*, *Staphylococcus aureus*, *Proteus mirabilis*, *Escherichia coli*, and *Psudomonas aeruginosa.* [40] Additionally, a novel nano-drug composed of GNPs and glycyrrhizin (GL) has been shown to effectively eliminate reactive oxygen/reactive nitrogen species (ROS/RNS) and damage-associated molecular patterns (DAMPs), thus attenuating M1 polarization of macrophages, and enhancing the abundance and diversity of beneficial probiotics [41]. Interestingly, GNPs can also modulate gut microbiota by impacting host immune responses. Studies have revealed that GNPs were capable of activating immune cells, triggering the production of cytokines and other inflammatory mediators, thus indirectly influencing the composition and functionality of gut microbiota [42, 43]. Furthermore, recent studies suggested that oral administration of chiral GNPs could reshape the gut microbiome composition in Alzheimer’s disease (AD) mice, manifested by a significant increase in *Lactobacillus* and *Clostridium.* Meanwhile, fecal metabolite analysis showed that chiral GNPs intervention dramatically altered the tryptophan metabolic pathway, which increased the level of gut metabolite (indole-3-acetic acid), thereby reducing neuroinflammation and improving the cognitive ability of AD mice [44]. Notably, a number of comprehensive studies have demonstrated that GNPs possessed prominent anti-angiogenic, anti-inflammatory, and osteogenic effects, which contributed to significant alleviation in inflammatory responses and improved excessive bone loss during the progression of OP and RA [9, 45, 46]. Based on these, it is reasonable to speculate that GNPs could modulate the composition of gut microbiota and its metabolites, and restrain inflammatory responses, thus retarding the pathologic progression of OA via the “microbiota-gut-joint” axis.

## Materials and methods

### Reagents

All reagents used in this experiment were analytical grade and could be used directly without further purification. The GNPs were obtained from Wuhan MICE Biotechnology Co. Ltd, and were synthesized by using the classic citrate-based reduction method [47]. Details regarding the specific preparation method can be found in the Supplementary Materials and Methods section. GNPs samples were diluted in water by ultrasonic dispersion, and the zeta potential was measured by Zetasizer Nano ZS90. Additionally, the morphological characteristics of GNPs (including the size distribution and homogeneity) were analyzed by using a scanning electron microscope (Hitachi Regulus SU8100). The specific process is as follows. First, the GNPs samples were diluted and placed onto the carbon-coated copper grid, and then naturally dried at room temperature, as well as subsequently stained with 2% uranyl acetate. Micrographs of samples were obtained using backscattered electron (BSE) detectors at 10 or 15 kV and 30 Pa. Finally, the size of the mean nanoparticle is selected as the sample size in this experiment.

### Animal studies

Several C57BL/6J mice (8 weeks, male, 20–25 g) were obtained from Hunan SJA Laboratory Animal Co.Ltd. All experimental mice were maintained under specific pathogen-free (SPF) conditions with a 12-h light-dark cycle, and had free access to food and water. All male C57BL/6J mice were used for experiments based on the current ethical regulations for animal care and use in China and were approved by Army Medical University (No. AMUWEC20232385). All experimental male C57BL/6J mice underwent surgical procedures and suturing after anesthesia to induce the OA models of anterior cruciate ligament transection (ACLT) as previously described [48]. Next, mice were randomly assigned to six experimental groups: ACLT mice treated with vehicle (namely ACLT^Vehicle^, *n* = 7), ACLT mice treated with GNPs (namely ACLT^GNPs^, *n* = 7), ACLT mice treated with antibiotics (ABX) and vehicle (namely ACLT^ABX+Vehicle^, *n* = 10), ACLT mice treated with ABX and GNPs (namely ACLT^ABX+GNPs^, *n* = 10), ACLT mice that underwent the operation of fecal microbiota transplantation (FMT) from ACLT^Vehicle^ group (namely FMT^ACLT+Vehicle^, *n* = 5), and ACLT mice that underwent the operation of FMT from ACLT^GNPs^ group (namely FMT^ACLT+GNPs^, *n* = 5). Three days after ACLT surgery, ACLT^GNPs^ mice were given continuous gavage with GNPs at a dose of 0.01 mg/g bw/day for 8 weeks. The control groups were given continuous gavage with an equivalent amount of normal saline.

Before ACLT surgery, ACLT^ABX+Vehicle^ and ACLT^ABX+GNPs^ mice received broad-spectrum ABX treatment for 7 days, including Vancomycin, 100 mg/kg bw/day (Cat#: L2103032, aladdin); Neomycin (sulfate), 200 mg/kg bw/day (Cat#: HY-B0470/CS-2584, MedChemExpress); Metronidazole, 200 mg/kg bw/day (Cat#: E2209095, aladdin); and Ampicillin Na, 200 mg/kg bw/day (Cat#: I2110038, aladdin). For the next 8 weeks, a continuous ABX cocktail regimen as above described was administered orally every other day through the end of the study. The specific administration of the broad-spectrum ABX treatment via gavage is as follows: Firstly, prepare the broad-spectrum ABX solution with saline according to the aforementioned concentrations. Secondly, select an appropriately sized gavage needle (size 8) and syringe (1 ml) to minimize the risk of esophageal injury. Next, gently grasp the mouse by the scruff of its neck, ensuring not to apply excessive pressure. Then hold the gavage needle in dominant hand and insert it gently into the mouth of the mouse, aiming towards the esophagus. Slowly advance the gavage needle until the correct depth is achieved (that is usually indicated by the needle reaching the stomach without resistance). Administer the ABX solution slowly to prevent aspiration, then carefully withdraw the gavage needle. Monitor the mouse for a few minutes post-gavage to ensure there are no immediate adverse reactions, such as distress or difficulty breathing, which could indicate aspiration or injury. Finally, record the dosage, time, and any observations about mouse’s responses to the procedure.

To evaluate the effects of FMT on joints, we collected feces from mice after vehicle or GNPs treatment and performed FMT with the latest improved approach strictly [47, 49]. The specific operations are as follows. First, to eliminate gut microbiota, mice were treated with a cocktail of ABX for 7 days as described above. Subsequently, ACLT surgery was performed to set up the murine models of OA. Then, mice were randomly divided into two groups, namely FMT^ACLT+GNPs^ and FMT^ACLT+Vehicle^. Next, the above two groups were administered orally with fecal suspensions (feces dissolved in saline, 200 µl/mouse) every other day, which were respectively obtained from the ACLT^GNPs^ group and the ACLT^Vehicle^ group. All experimental mice were euthanized on the 56th day after ACLT surgery, and their knee joints were taken for follow-up analysis. Additionally, feces samples were harvested for further subsequent analyses. Specific operations are as follows: Fecal pellets (5–8) were delicately collected into individual sterile tubes and swiftly fixed upon the dry ice ensuring immediate preservation of their microbial content. Subsequently, fecal specimens were immediately stored at a frigid temperature of -80℃ until analysis.

### Micro-CT analysis

All bilateral knee joints from experimental mice were obtained and fixed for 72 h at 4℃ by using 4% polyoxymethylene. Whole knee joints were scanned and reconstructed by using the Bruker Micro-CT Skyscan 1272 system (Kontich, Belgium). The isotropic voxel size was used as 7.0 μm. The software for reconstruction as well as for processing and analysis are Nrecon (Ver.1.6.10, Kontich, Belgium) and CT analyzer (Kontich, Belgium) respectively. The subchondral bone regions of the distal femur and proximal tibia were selected as the region of interest (ROI), and 3D model visualization software (CTvox, Bruker micro-CT, ver.3.3.1.0) was used for 3-dimensional histomorphometric analysis. The following parameters were quantitatively analyzed: bone mineral density (BMD), bone volume fraction (BV/TV), trabecular bone thickness (Tb.Th), trabecular number (Tb.N), and trabecular bone separation (Tb.Sp) [50]. 

### Histological and immunohistochemistry (IHC) analysis

The knee joints were fixed with 4% paraformaldehyde for 48 h, and followed by decalcification in 10% ethylenediaminetetraacetic acid (EDTA) for 2 weeks. Then samples were embedded with paraffin and serially sectioned into the thickness of 5 μm frontal slices. Subsequently, every knee joint slice was stained sequentially with Hematoxylin-eosin (H&E), Safranin-O, and Toluidine Blue according to regular procedures [51]. The histopathological changes in osteoarthritic cartilage were evaluated by using the Osteoarthritis Research Society International (OARSI) grading system and MANKIN scores [52]. In order to assess the side effects of GNPs, liver and kidney tissue samples were fixed, embedded, sectioned, and stained with H&E as described above, followed by further histological analysis. For immunohistochemical staining, sections blocked by 5%BSA were incubated with the corresponding primary antibodies: anti-Aggrecan (ABclonal, Cat#: A11691), anti-MMP13 (Proteintech, Cat#: 18165-1-AP), and overnight at 4℃. Then, they were treated with the corresponding secondary antibodies (Santa Cruz Biotechnology, Santa Cruz, CA) for 1 h. The relative expressions of positive staining were evaluated by Image J software.

### Immunofluorescence assay

Immunofluorescence staining was performed separately to validate the percentage and changes in the number of M1 subtype and M2 subtype macrophages in each group. In brief, bone sections were incubated with individual primary antibodies to mouse F4/80 (Biolegend, Cat#: 123,105), CD86 (ABclonal, Cat#: A16805), and CD206 (ABclonal, Cat#: A8301) overnight at 4℃. M1 subtype macrophages and M2 subtype macrophages were identified as F4/80^+^ and CD86^+^ cells, as well as F4/80^+^ and CD206^+^ cells, respectively. Subsequently, secondary antibodies conjugated with fluorescence were used at room temperature for 1 h while avoiding light. The nuclei were counterstained with DAPI, and fluorescence analysis was performed with a Leica TCS SP8 confocal microscope.

Additionally, every knee joint slice was blocked by 5%BSA and incubated with the corresponding primary antibodies: anti-TRAP (Bioss, Cat#: bs-16578R), anti-CGRP (Bioss, Cat#: bs-0791R), anti-Netrin-1 (Bioss, Cat#: bs-1858R), and overnight at 4℃.

### Fecal 16 S rRNA gene sequencing

DNA from samples of ACLT^Vehicle^ and ACLT^GNPs^ groups was extracted using the CTAB in the light of the instructions of the manufacturer. The total DNA was eluted in 50 µL of Elution buffer and stored at -80℃ for PCR measurement via Biotree Biomedical Technology Co., Ltd., (Shanghai, China). The hypervariable region V3-V4 of the bacterial 16S rRNA gene were amplified with primer pairs 341F (5’-CCTACGGGNGGCWGCAG-3’) and 805R (5’-GACTACHVGGGTATCTAATCC-3’). The PCR products were purified by AMPure XT beads (Beckman Coulter Genomics, Danvers, MA, USA) and quantified by Qubit (Invitrogen, USA). Amplicon pools were generated for sequencing, and the size of the amplicon libraries was evaluated on the Agilent 2100 Bioanalyzer (Agilent, USA), as well as the quantity of the amplicon libraries was assessed on the Library Quantification Kit for Illumina (Kapa Biosciences, Woburn, MA, USA). The library sequencing was performed on the NovaSeq PE250 platform according to the manufacturer’s recommendations. Details of the analysis methods were provided in the Supplementary Materials and Methods.

### Fecal SCFA metabolomics analysis

Take fecal samples into the 2 mL EP tubes, which were extracted with 1 mL H_2_O. The extract was then homogenized in a ball mill, and centrifuged to obtain the supernatant into a fresh 2 mL EP tubes. Add 0.1 mL 50% H_2_SO_4_ and 0.8 mL of extracting solution (25 mg/L stock in methyl tert-butyl ether) as internal standard, and then the supernatant was extracted for GC-MS analysis in accordance with the protocol of the manufacturer (Biotree Biomedical Technology Co., Ltd., Shanghai, China). GC-MS analysis was performed using SHIMADZU GC2030-QP2020 NX gas chromatography-mass spectrometer coupled with Agilent HP-FFAP capillary column (30 m×250 μm×0.25 μm, J&W Scientific, Folsom, CA, USA). Details of the method are provided in the Supplementary Materials and Methods.

### Gut permeability analysis

In order to assess the intestinal permeability, paraformaldehyde-fixed and paraffin-embedded sections of mouse colon tissue were used for immunostaining as previously described [53]. Specifically, the sections were first blocked for 0.5 h with 5% BSA at room temperature and then incubated with primary antibodies specific for ZO-1 (Bioss, Cat#: bs-1329R) and Occludin (Bioss, Cat#: bs-10011R) overnight at 4 °C. Next, these sections were conjugated with the fluorescently labeled secondary antibody (Abcam, UK) (1:50 dilution) for 1 h at 37℃ in the dark. Finally, a Leica TCS SP8 confocal microscope was performed to examine and acquire images. Besides, all images were analyzed by Image J software.

### Multiple inflammatory cytokines array

At the scheduled time, mice were subjected to a 12-hour fasting period before being anesthetized for blood collection via the retro-orbital sampling [54]. Subsequently, the collected whole blood was allowed to fully clot at room temperature for 1 h, after which it was subjected to centrifugation at 4000 rpm/min (4℃) for 15 min to obtain serum, which was then stored at -80℃ until sample preparation and analysis. The inflammatory cytokines and chemokines were detected by a Luminex protein biochip testing system (Bio-Plex MAGPIX System, Bio-Rad) with a test kit (Bio-Plex Pro Mouse Cytokine Grpl Panel 23-plex, Wayen Biotechnologies, Shanghai) according to the manufacturer’s instructions. Briefly, the serum samples were incubated in 96-well plates embedded with microbeads for 1 h followed by incubation with detection antibody for 30 min. Subsequently, streptavidin-PE was added to each well for 10 min and values were read using the Bio-Plex MAGPIX System (Bio-Rad).

### Statistical analysis

In this study, all the data are presented as mean ± standard deviation. Statistical analyses were performed using Prism GraphPad Prism software (version 6.02). Statistical significance was calculated using Unpaired Student’s t-test for two groups for bone histomorphometry index, IHC and IF results. Welch’s correction was applied when the F test was significant. Wilcoxon rank-sum test was used for bacterial taxonomic analyses. Correlations were assessed using Spearman’s rank correlation test. *p* < 0.05 was considered to indicate a significant difference and is shown in the figures. Asterisks used to indicate significance corresponds to the following: *, *p* < 0.05; **, *p* < 0.01; ***, *p* < 0.001; ****, *p* < 0.0001. N.S represented not significant.

## Results

### The preparation and characterization of GNPs

**Fig. 1:**
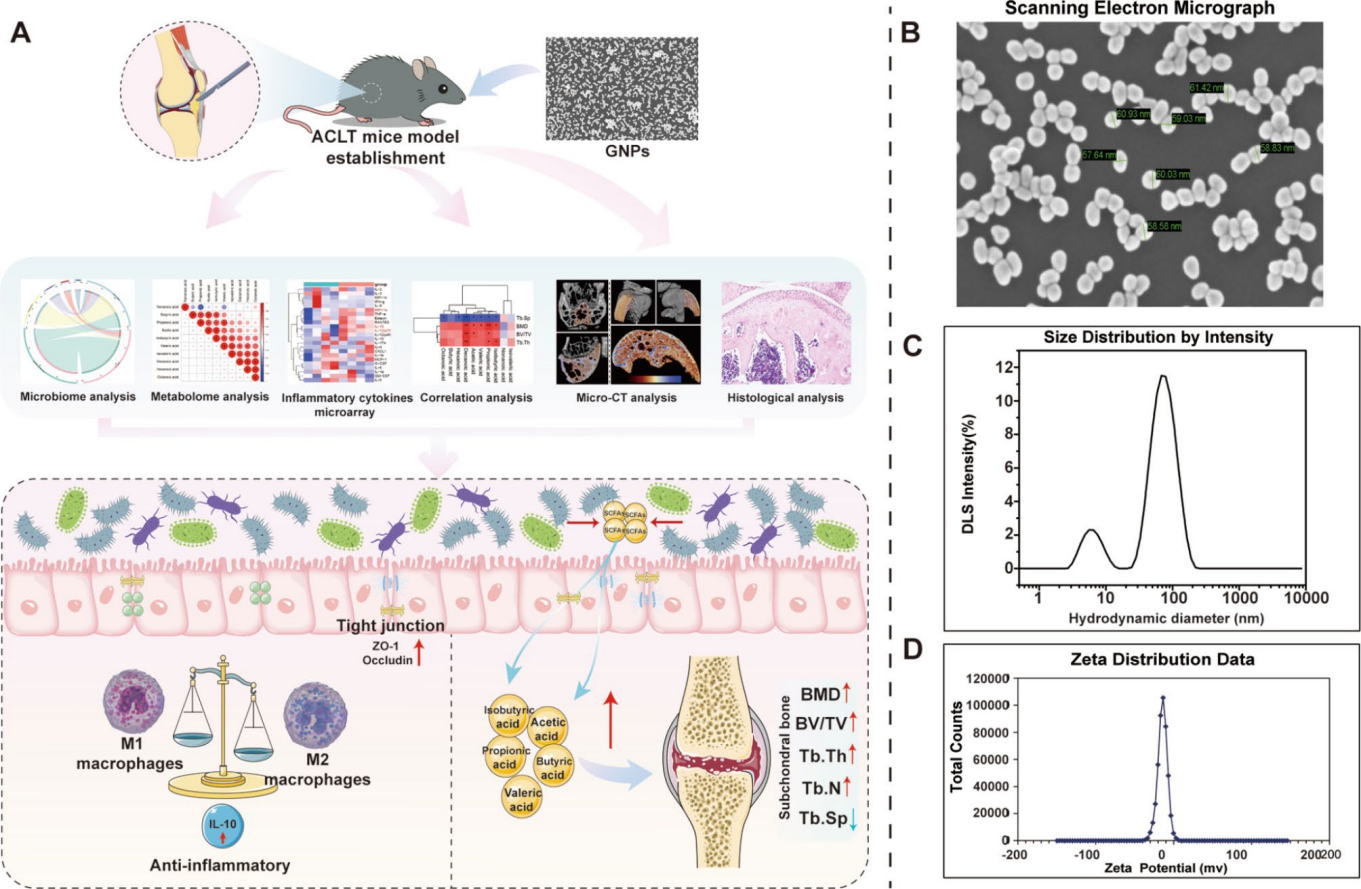
The characterization and main experimental design of GNPs. (**A**) The detailed experimental design and analytical approach of the study. (**B**) The general characterization of GNPs under scanning electron microscope. (**C**) Size distribution of GNPs. (**D**) Zeta potential distribution of GNPs

As is shown in Fig. 1A, we exhibited the main experimental design and methods, which contributed to the comprehensive exploration and understanding of our research. The specific preparation method of GNPs can be found in the Supplementary Materials and Methods. Upon evaluation using a scanning electron microscope, the GNPs exhibit excellent uniformity with an average diameter of approximately 60 nm (Fig. 1B and C). The zeta potential of the synthesized GNPs was characterized to assess their surface charge and colloidal stability. The values of zeta potential were approximately −14 mv (Fig. 1D).

### GNPs significantly ameliorated cartilage matrix degradation and subchondral bone loss in a murine OA model

**Fig. 2:**
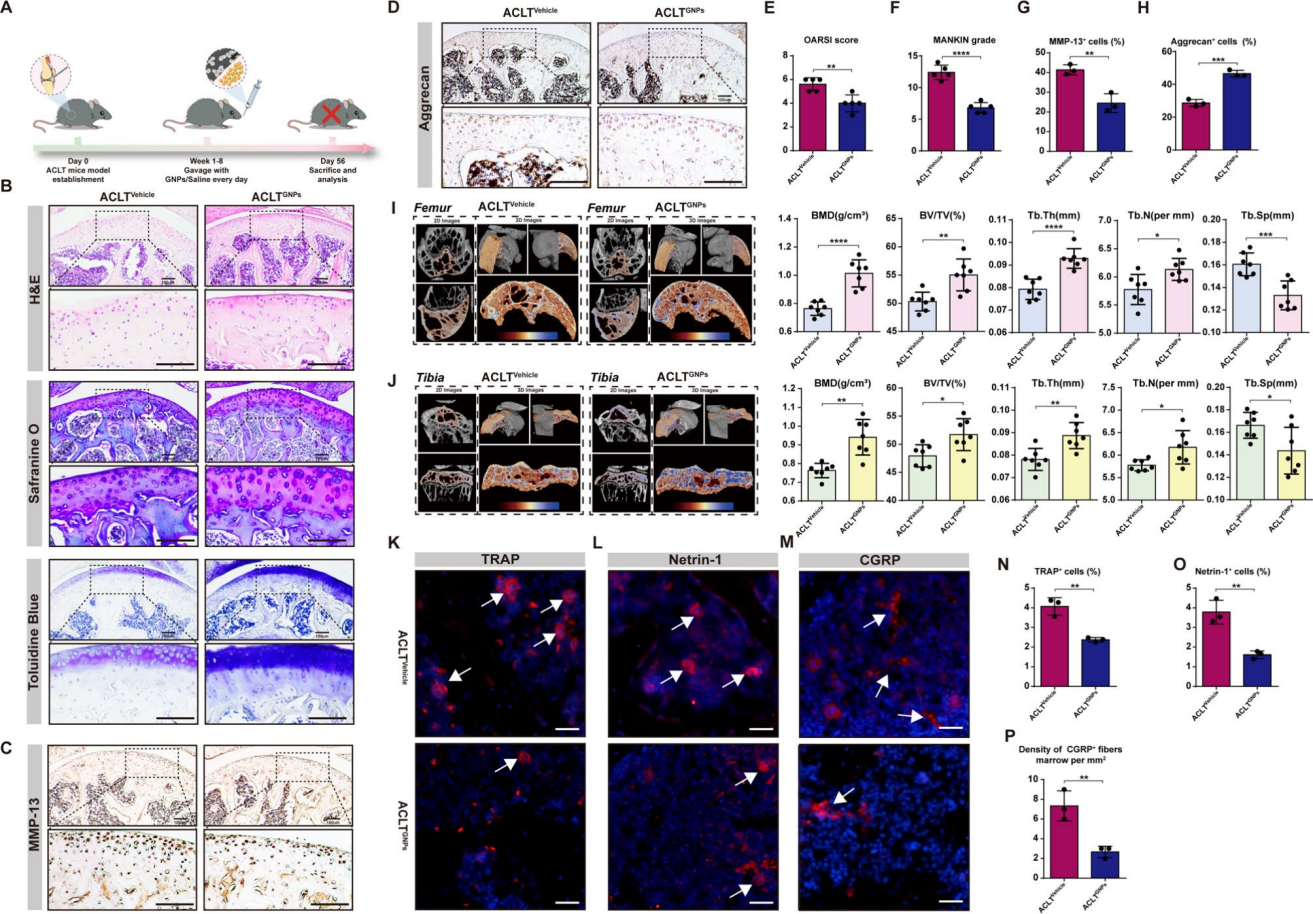
GNPs treatment alleviated ACLT-induced OA. (**A**) To evaluate the mitigation of GNPs on OA, C57BL/6J mice (6–8 weeks, male, 20–25 g) were selected to undergo anterior cruciate ligament transection surgery to induce the OA models, and randomly assigned to ACLT^Vehicle^ or ACLT^GNPs^ group. The ACLT^Vehicle^ and ACLT^GNPs^ groups were given continuous gavage with the equivalent amount of saline and GNPs for 8 weeks, respectively. (**B**) H&E, Safranine O and Toluidine Blue staining of femur articular cartilage in ACLT^Vehicle^ and ACLT^GNPs^ groups. (**C**) Immunohistochemical staining of the expression of MMP-13 in articular cartilage of sections. (**D**) Immunohistochemical staining of the expression of Aggrecan in articular cartilage of sections. (**E**) Quantitative analysis of Osteoarthritis Research Society International (OARSI) histology scoring. (**F**) Quantitative analysis of MANKIN grading. (**G**) Quantitative analysis of percentage of cells with positive staining for MMP-13 in articular cartilage. (**H**) Quantitative analysis of percentage of cells with positive staining for Aggrecan in articular cartilage. (**I**) Representative µCT images about 2D images of longitudinal section femurs and 3D images of distal femur. The coloring parts represent the regions of interest (ROI) for analysis. Quantification of femoral BMD, BV/TV, Tb.Th, Tb.N and Tb.Sp in indicated groups, *n* = 7. (**J**) Representative µCT images about 2D images of longitudinal section tibias and 3D images of proximal tibias. The coloring parts represent the ROI for analysis. Quantification of tibial BMD, BV/TV, Tb.Th, Tb.N and Tb.Sp in indicated groups, *n* = 7. (**K**) Representative images of IF staining of TRAP in the subchondral bone of the ACLT^Vehicle^ and ACLT^GNPs^ mice. (**L**) Representative images of IF staining of Netrin-1 in the subchondral bone of the ACLT^Vehicle^ and ACLT^GNPs^ mice. (**M**) Representative images of IF staining of CGRP^+^ fibers in the subchondral bone of the ACLT^Vehicle^ and ACLT^GNPs^ mice. (**N**) Quantitative analysis of TRAP staining. (**O**) Quantitative analysis of Netrin-1staining. (**P**) Quantitative analysis of CGRP^+^ fibers staining. (*p < 0.05, **p < 0.01, ***p < 0.001, ****p < 0.0001, N.S represented not significant)

ACLT-induced murine OA model is a widely used OA model that could effectively mimic the typical pathological features of human OA, such as cartilage degradation, subchondral change, and synovitis [55–57]. Therefore, we employed the ACLT-induced OA mice model to investigate the role of GNPs in OA progression. As is shown in Fig. 2A, ACLT mice were randomly divided into two groups and treated with GNPs at a dose of 0.01 mg/g bw/day and the same volume of saline via gastric gavage for 8 weeks, respectively. GNPs are non-toxic to the liver and kidney of mice based on H&E staining (Fig.  S1). At a later time point of 8 weeks post-ACLT surgery, we have collected articular samples and performed H&E, Safranine O, and Toluidine Blue staining to measure the degradation of articular cartilage. These results demonstrated that GNPs administration markedly reversed the cartilage alteration and the loss of proteoglycan, resulting in a significant reduction in the OARSI scores and MANKIN grades (Fig. 2B, E and F). Additionally, cartilage extracellular matrix anabolic marker Aggrecan and catabolic marker MMP-13 were evaluated via IHC staining. The staining results of the uncalcified cartilage layer showed that the proportion of MMP-13 positive cells was notably decreased while the percentage of Aggrecan positive cells was increased in the ACLT^GNPs^ group compared with ACLT^vehicle^, which exhibited the protective effect of GNPs on cartilage degradation in OA (Fig. 2C, D, G, and H).

It is well known that the subchondral bone beneath the cartilage layer in joints plays a crucial role in the progression of OA. The release of factors from the subchondral bone and the interaction between the OCs and chondrocytes can promote a chronic cycle of inflammation and tissue damage within the joint [3]. Emerging research has revealed the deterioration of subchondral bone disrupts the delicate balance of structure and distribution of forces within the joint, leading to a cascade of events that perpetuate the progression of cartilage damage [58, 59]. Therefore, to explore whether GNPs could improve OA progression by reducing subchondral bone loss, we reconstructed 2D and 3D images of the subchondral bone of the femur and tibia by using µCT. The representative images of µCT provided detailed visualization of the morphology of the subchondral bone, showing the improvement of subchondral bone loss in femur and tibia. Quantification analyses demonstrated that GNPs markedly increased BMD (*p* < 0.0001), BV/TV (*p* = 0.0024), Tb.Th (*p* < 0.0001), and Tb.N (*p* = 0.0147), and reduced Tb.Sp (*p* = 0.0008) in the subchondral bone of femur (Fig. 2I). In consistent with the alteration in the femur, the tibial subchondral bone exhibited the same tendency. BMD (*p* = 0.0019), BV/TV (*p* = 0.0133), Tb.Th (*p* = 0.0030), and Tb.N (*p* = 0.0293) were increased and Tb.Sp (*p* = 0.0273) was decreased significantly in the ACLT^GNPs^ group (Fig. 2J).

Additionally, *Shouan Zhu et al.* elucidated a novel pathophysiological mechanism in OA, whereby OCs within the subchondral bone contribute to nociceptive innervation. They demonstrated that OCs facilitated the extension of sensory nerve axons by secreting the axonal guidance molecule Netrin-1, subsequently exacerbating OA-related pain and propelling the progression of OA [60]. Utilizing immunofluorescent staining techniques, it was observed that administration of GNPs substantially attenuated the abundance of tartrate-resistant acid phosphatase (TRAP)-positive OCs in the subchondral bone following ACLT surgery compared to vehicle-treated controls, as depicted in Fig. 2K and N. In concordance with these findings, the expression of Netrin-1, as well as calcitonin gene-related peptide (CGRP)-positive sensory neurons, which were implicated in mediating OA-related pain, was significantly reduced in the subchondral bone marrow of ACLT^GNPs^ mice, as demonstrated in Fig. 2L, M, O, and P. These findings demonstrated that GNPs could effectively improve cartilage matrix degradation and subchondral bone remodeling in the ACLT-induced OA model.

### Antibiotic administration reversed GNPs-mediated protective effects on OA progression

**Fig. 3:**
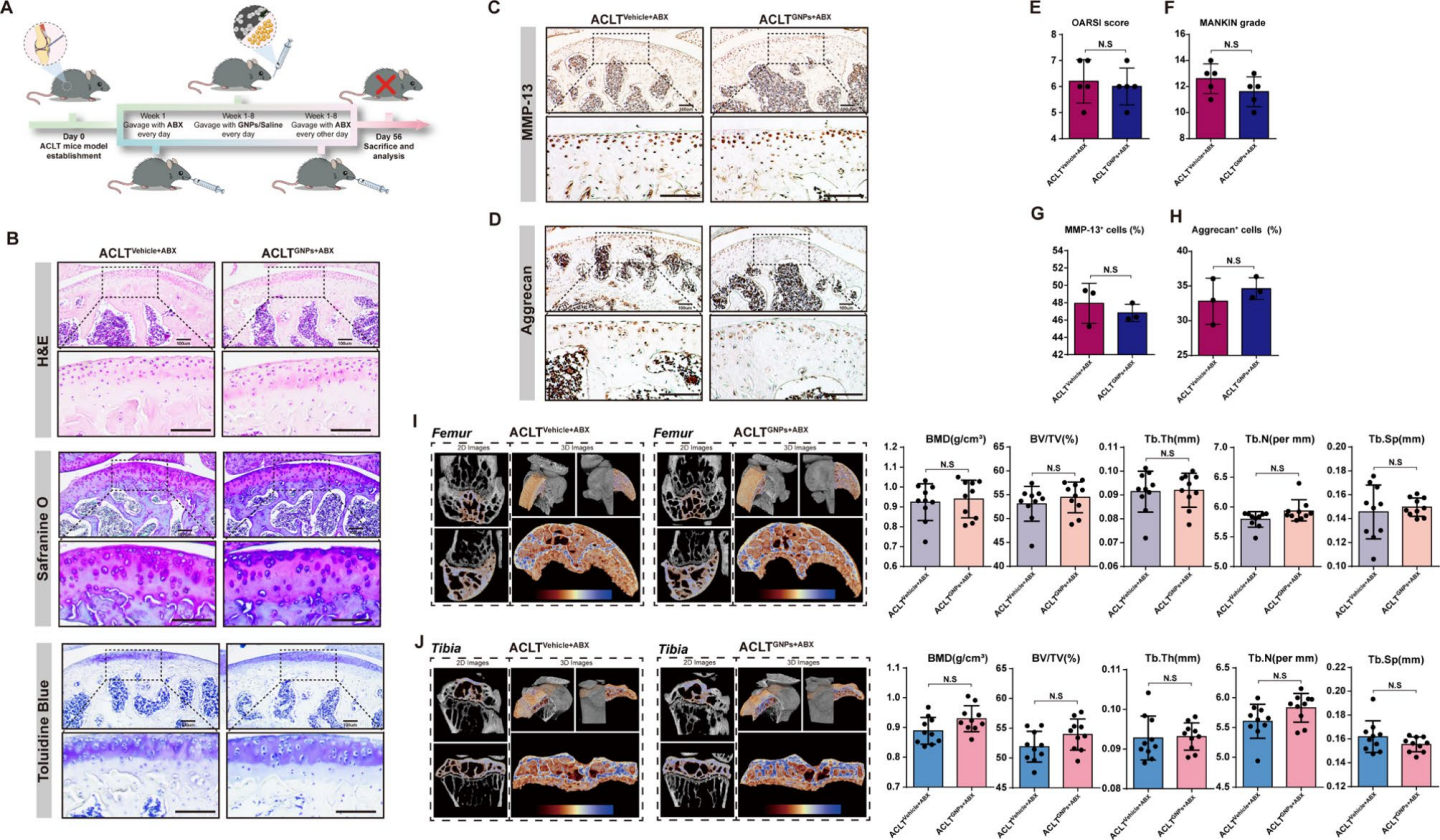
The protective effect of GNPs against ACLT-induced OA disappeared after gut microbiota consumption. (**A**) The schematic diagram of gut microbiota consumption experiments. (**B**) H&E, Safranine O and Toluidine Blue staining of femur articular cartilage in ACLT^Vehicle+ABX^ and ACLT^GNPs+ABX^ groups. (**C**) Immunohistochemical staining of the expression of MMP-13 in articular cartilage of sections. (**D**) Immunohistochemical staining of the expression of Aggrecan in articular cartilage of sections. (**E**) Quantitative analysis of OARSI histology scoring. (**F**) Quantitative analysis of MANKIN grading. (**G**) Quantitative analysis of percentage of cells with positive staining for MMP-13 in articular cartilage. (**H**) Quantitative analysis of percentage of cells with positive staining for Aggrecan in articular cartilage. (**I**) Representative µCT images about 2D images of longitudinal section femurs and 3D images of distal femurs. The coloring parts represent the ROI for analysis. Quantification of femoral BMD, BV/TV, Tb.Th, Tb.N and Tb.Sp in indicated groups, *n* = 10. (**J**) Representative µCT images about 2D images of longitudinal section tibias and 3D images of proximal tibias. The coloring parts represent the ROI for analysis. Quantification of tibial BMD, BV/TV, Tb.Th, Tb.N and Tb.Sp in indicated groups, *n* = 10. (*p < 0.05, **p < 0.01, ***p < 0.001, ****p < 0.0001, N.S represented not significant)

The etiology of OA is multifaceted and remains incompletely elucidated, yet accumulating evidence underscores the gut microbiota as a pivotal player in OA pathogenesis. The complex interplay between the gut microbiome and host is believed to influence OA progression through various mechanisms including modulation of local and systemic inflammation, alteration of metabolic profiles, and impact on joint cartilage homeostasis [14]. To verify whether GNPs ameliorated ACLT-induced OA progression in a gut microbiota-dependent manner, we performed antibiotic cocktail treatment on ACLT mice to construct a pseudo-germ-free (PGF) murine model. Specifically, ACLT mice were randomly separated into ACLT^GNPs+ABX^ and ACLT^Vehicle+ABX^ groups, both of which received ABX treatment, and simultaneously, the ACLT^GNPs+ABX^ group was orally administered with GNPs, while the ACLT^Vehicle+ABX^ group received an equivalent amount of saline (Fig. 3A). Interestingly, we found that ABX abolished GNPs-mediated cartilage matrix protective effects. No significant differences were observed between the two groups in terms of H&E, Safranin O, and Toluidine Blue staining (Fig. 3B). Additionally, there were no differences in the OARSI scores and MANKIN grades (Fig. 3E and F). These results correspond with the IHC staining of aggrecan and MMP-13, with the almost indistinguishable proportion of MMP-13 and aggrecan-positive cells in the uncalcified cartilage layer (Fig. 3C, D, G and H). Furthermore, the administration of an antibiotic cocktail nullified the advantageous effects of GNPs on the subchondral bone architecture. This was evidenced by the corresponding alterations in BMD, BV/TV, Tb.Th, Tb.N, and Tb.Sp, which were neutralized in the absence of gut microbiota (Fig. 3I and J). Collectively, these results revealed that GNPs attenuated ACLT-induced OA progression in a gut microbiota-dependent manner.

### Fecal microbial transplantation (FMT) mitigated OA progression

**Fig. 4:**
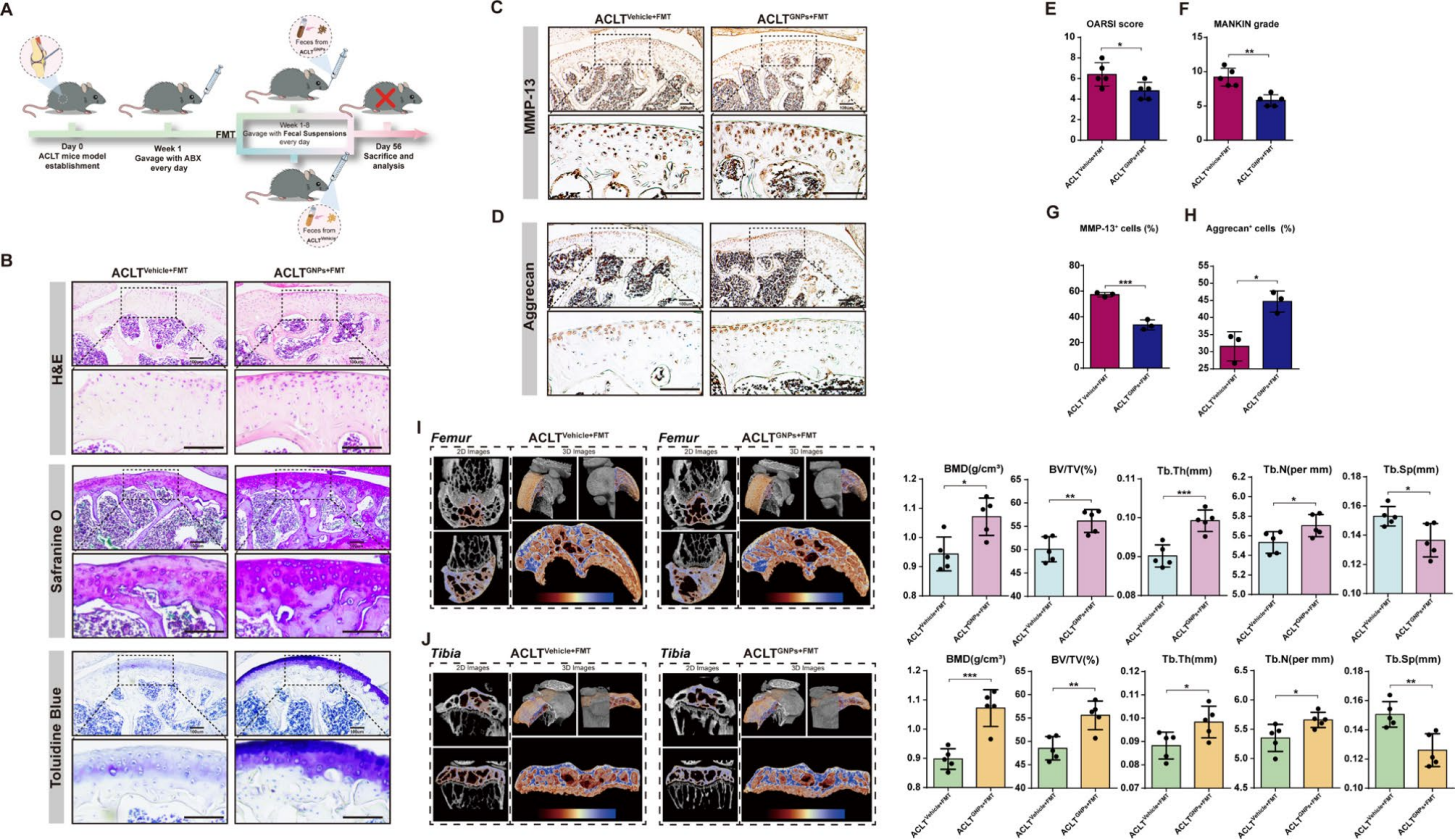
Fecal microbiota transplantation alleviated ACLT-induced OA. (**A**) The schematic diagram of the FMT experiments. (**B**) H&E, Safranine O and Toluidine Blue staining of femur articular cartilage in ACLT^Vehicle+FMT^ and ACLT^GNPs+FMT^ group. (**C**) Immunohistochemical staining of the expression of MMP-13 in articular cartilage of sections. (**D**) Immunohistochemical staining of the expression of Aggrecan in articular cartilage of sections. (**E**) Quantitative analysis of OARSI histology scoring. (**F**) Quantitative analysis of MANKIN grading. (**G**) Quantitative analysis of percentage of cells with positive staining for MMP-13 in articular cartilage. (**H**) Quantitative analysis of percentage of cells with positive staining for Aggrecan in articular cartilage. (**I**) Representative µCT images about 2D images of longitudinal section femurs and 3D images of distal femurs. The coloring parts represent the ROI for analysis. Quantification of femoral BMD, BV/TV, Tb.Th, Tb.N and Tb.Sp in indicated groups, *n* = 5. (**J**) Representative µCT images about 2D images of longitudinal section tibias and 3D images of proximal tibias. The coloring parts represent the ROI for analysis. Quantification of tibial BMD, BV/TV, Tb.Th, Tb.N and Tb.Sp in indicated groups, *n* = 5. (*p < 0.05, **p < 0.01, ***p < 0.001, ****p < 0.0001, N.S represented not significant)

FMT is a novel and effective method that involves the transplantation of fecal microbiota from a healthy donor into the digestive tract of a patient with a compromised gut microbiome, which has been widely applied in the treatment of patients with chronic gastrointestinal infections and inflammatory bowel diseases [61]. To further determine the participation of gut microbiota in the protective effects of GNPs on OA, fecal samples were collected from ACLT^GNPs^ and ACLT^Vehicle^ mice and then transplanted into gut microbiota-depleted mice that underwent ACLT surgery, resulting in two groups referred to as ACLT^GNPs+FMT^ and ACLT^Vehicle+FMT^ mice, respectively (Fig. 4A). This allowed us to examine the impact of the respective gut microbiota on the OA progression. Surprisingly, fecal microbiota from ACLT^GNPs^ mice markedly relieved ACLT-induced cartilage matrix degradation and enhanced related synthesis compared with that from ACLT^Vehicle^ mice. The staining results exhibited lower degradation and loss of cartilage components and better structural integrity of the tissue in ACLT^GNPs+FMT^ mice (Fig. 4B, E and F). Consistently, a significant reduction in the percentage of MMP-13 positive chondrocytes, alongside an increase in Aggrecan-positive cell populations, was observed in the cartilage tissue following administration of fecal samples derived from ACLT^GNPs^ mice (Fig. 4C, D, G, and H), which added further evidence to the premise that gut microbiota from the ACLT^GNPs^ group exerted a protective effect against the degradation of the cartilaginous matrix. These alterations in cellular composition underscore the potential regulatory role of the transplanted fecal microbiota in maintaining cartilage homeostasis and integrity. Histomorphometry indexes of subchondral bone including BMD (*p* = 0.0108), BV/TV (*p* = 0.0059), Tb.Th (*p* = 0.0009), Tb.N (*p* = 0.0414), and Tb.Sp (*p* = 0.0240) exhibited a remarkable amelioration in the ACLT^GNPs+FMT^ group (Fig. 4I and J). These results further demonstrated that GNPs ameliorated subchondral bone loss and mesochondrium degradation in a gut-microbiota manner.

### GNPs supplementation reprogramed the structure and combination of gut microbiota

**Fig. 5:**
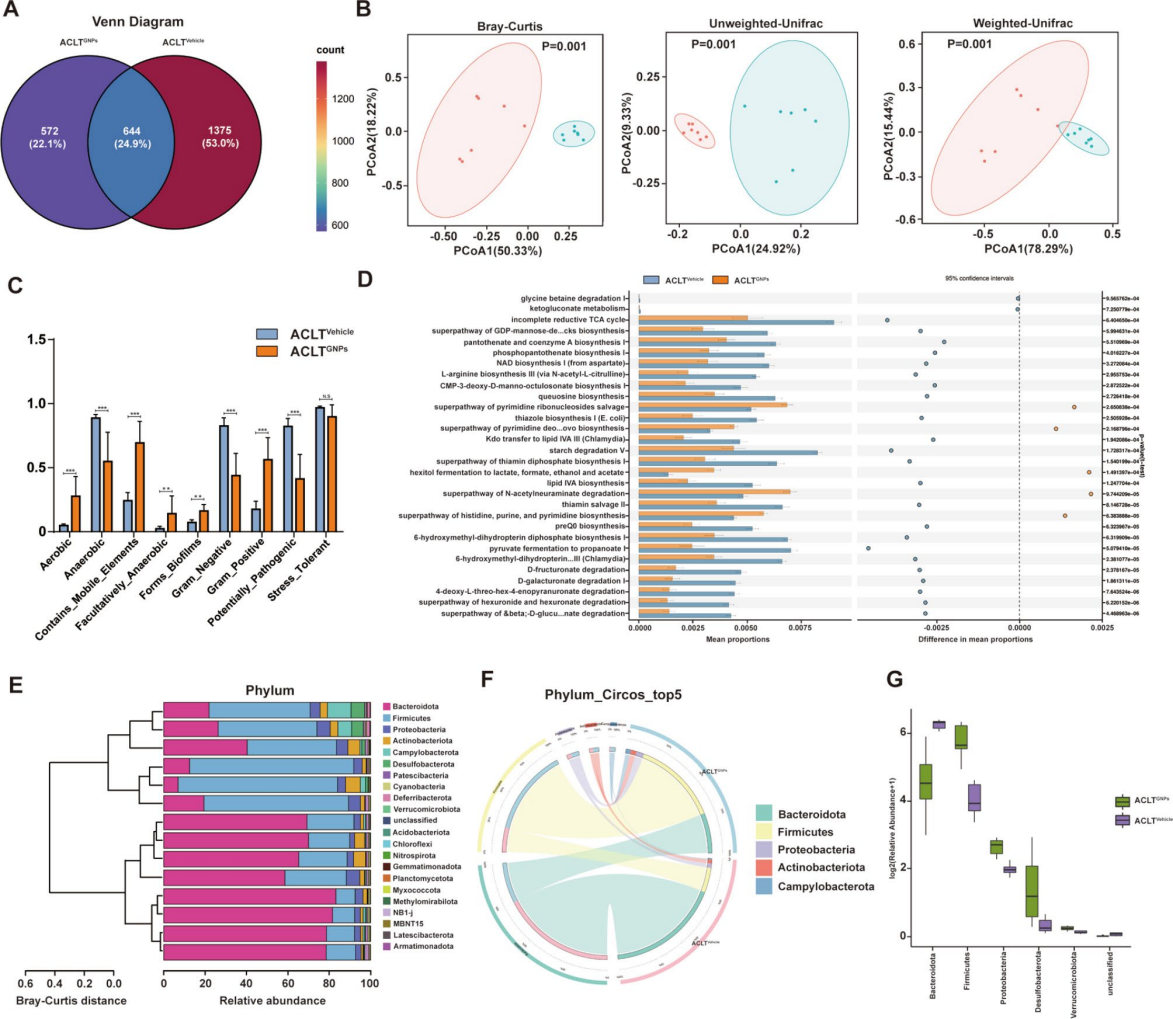
GNPs treatment significantly changed gut microbiota diversity and composition. (**A**) Venn diagram of features in ACLT^Vehicle^ and ACLT^GNPs^ groups. (**B**) PCoA based on the Bray-Curtis distance, Unweighted-UniFrad and Weighted-UniFrad distances. (**C**) Bar graphs about the abundance of microbiota with functional clustering in ACLT^Vehicle^ and ACLT^GNPs^ groups. (**D**) Metabolic potential of microbial taxa predicted by PICRUSt2. The figure illustrates the relative abundance of microbial groups and their roles in major metabolic pathways, including carbohydrate, amino acid, and lipid metabolism. (**E**) Bar graphs about gut microbiota at phylum taxonomic level in ACLT^Vehicle^ and ACLT^GNPs^ groups. (**F**) Circos plot about the relative abundance of bacterial phyla between ACLT^Vehicle^ and ACLT^GNPs^ groups. The different colored ribbon represents specific phylum and the width of ribbon is directly proportional to the abundance of phylum. The ribbon connects bacterial taxa to their respective sample. (**G**) Statistical differences of gut microbiota at phylum taxonomic level between ACLT^Vehicle^ and ACLT^GNPs^ groups were evaluated with box plots. (A-G) *n* = 7 samples per group. (*p < 0.05, **p < 0.01, ***p < 0.001, ****p < 0.0001, N.S represented not significant)

Given that gut microbiota has been implicated in the progression of OA and mediates GNPs-related anti-osteoarthritis effects, we performed high-throughput gene-sequencing analysis of 16S rDNA in fecal bacterial DNA isolated from ACLT^GNPs^ and ACLT^Vehicle^ mice. As shown in Fig. 5A, the Venn diagram illustrated the feature diversity of gut microbiota between ACLT^GNPs^ and ACLT^Vehicle^ groups. Specifically, there are 644 similar features present in both two groups, accounting for 24.9% of their total features. Meanwhile, group ACLT^GNPs^ and ACLT^Vehicle^ each harbored 572 and 1375 unique features, comprising 22.1% and 53.0% of the total features, which suggested notable differences in their microbial composition. These features were shared by 25 phyla, 58 classes, 130 orders, 200 families, 362 genera, and 459 species. Subsequent analyses were conducted to delve into the diversity and dissimilarities of gut microbiota between ACLT^GNPs^ and ACLT^Vehicle^ groups, building upon the species annotation and aforementioned characteristics.

We initially performed alpha diversity analyses of gut microbiota to evaluate the within-sample diversity of the microbial community using a generalized linear model through different methodologies. The observed plateau in the rarefaction curves demonstrated that the sampling efforts have adequately captured the majority of the microbial diversity, which proved the comprehensiveness of the analysis (Fig. S2A). Related alpha diversity indexes such as Goods_coverage, Pielou_e, Shannon, and Simpson (*p* > 0.05) indicated no significant differences in microbial diversity, evenness, dominance, or coverage. Chao1 index (*p* < 0.05) was lower in the ACLT^GNPs^ group, which suggested that GNPs markedly decreased corresponding estimated richness (Fig. S2B). Subsequently, the beta diversity indices of the gut microbiota between two groups including Bray-Curtis (*p* = 0.001), Unweight-Unifrac (*p* = 0.001), Weight-Unifrac (*p* = 0.001), and Binary-Jaccard (*p* = 0.002) distances all demonstrated significantly dissimilar community compositions between the groups. The principal component analysis (PCA) supported these findings, further confirming distinct microbial profiles (Fig. 5B and S3A-B).

Additionally, we performed BugBase potential prediction to evaluate the phenotypic functions of microbial communities. Results demonstrated that GNPs administration significantly increased aerobic, containing mobile element, facultatively anaerobic, gram-positive and forming biofilm types, while decreased anerobic, gram-negative, and potentially pathogenic ones (Fig. 5C). To better illustrate the metabolic activities and relative abundance of specific microbial taxa in various environments, Phylogenetic Investigation of Communities by Reconstruction of unobobserved States 2 (PICRUSt2) was applicated for predicting their functional potential and exhibited multiple markedly different metabolic pathways including carbohydrate metabolism, amino acid metabolism, lipid metabolism, and various other biologically relevant pathways (Fig. 5D). We also performed Clusters of Orthologous Groups (COG) and KEGG analyses, which displayed various functional categories between the two groups (Fig. S3C-D).

As is shown in Fig. 5E, the microbial composition at the phylum level exhibited significant clustering patterns between ACLT^GNPs^ and ACLT^Vehicle^ groups, indicating that the samples within each group were highly similar and influenced by GNPs treatment. The Circos plot at the phylum level highlighted the dominance of five major bacteria, including *Bacteroidota*, *Firmicutes*, *Proteobacteria*, *Actinobacteriota* and *Campylobacterota* (Fig. 5F). In comparison with ACLT^Vehicle^ group, GNPs markedly upregulated the abundances of *Firmicutes, Proteobacteria, Desulfobacterota* and *Verrucomicrobiota*, while downregulate the abundance of *Bacteroidota* (Fig. 5G and S3E). Among these bacteria, *Firmicutes* and *Bacteroidetes* were the two most predominant phyla, and the ratio of F/B (*Firmicutes* to *Bacteroidetes*) has been implicated in the homeostasis of gut microbiota and serves as a hallmark of obesity, type 2 diabetes, and cardiovascular diseases [62, 63]. It is notable that F/B ratio was remarkably increased in ACLT^GNPs^ group (Fig. S3H). Specifically, *Bacteroidetes* abundance (75.25% vs. 26.58%, *p* = 0.0017) decreased while *Firmicutes* (16.26% vs. 56.56%, *p* = 0.0017) conspicuously increased with GNPs treatment (Fig. S3F-G). These findings further confirmed the dramatic alteration in the landscape of gut microbiota in ACLT^GNPs^ mice.

Subsequently, we observed changes in the relative abundance of key bacterial strains between ACLT^GNPs^ and ACLT^Vehicle^ groups at the class, order, and family levels.

At the class level, the ACLT^GNPs^ group showed a significant increase in *Bacilli* (*p* = 0.0017), *Gammaproteobacteria* (*p* = 0.0017), *Desulfovibrionia* (*p* = 0.0181), and *Coriobacteriia* (*p* = 0.0027), while a marked decrease in *Bacteroidia* (*p* = 0.0017) (Fig. S4A-4B). At the order level, we found that the relative abundances of *Lactobacillales* (*p* = 0.05), *Burkholderiales* (*p* = 0.0027), and *Coriobacteriales* (*p* = 0.004) were significantly higher, while *Bacteroidales* (*p* = 0.05) was lower in group ACLT^GNPs^ compared to group ACLT^Vehicle^ (Fig. S4C-4D). At the family level, *Lactobacillaceae* (*p* = 0.0017), *Sutterellaceae* (*p* = 0.0027), and *Eggerthellaceae* (*p* = 0.0027) exhibited a significant upward trend in ACLT^GNPs^ mice, and the level of *Prevotellaceae* (*p* = 0.0088) observably decreased after GNPs administration (Fig. S4E-4F). These results suggested that the ACLT^GNPs^ and ACLT^Vehicle^ groups may have completely different gut microbiota compositions at multiple taxonomic levels.

**Fig. 6:**
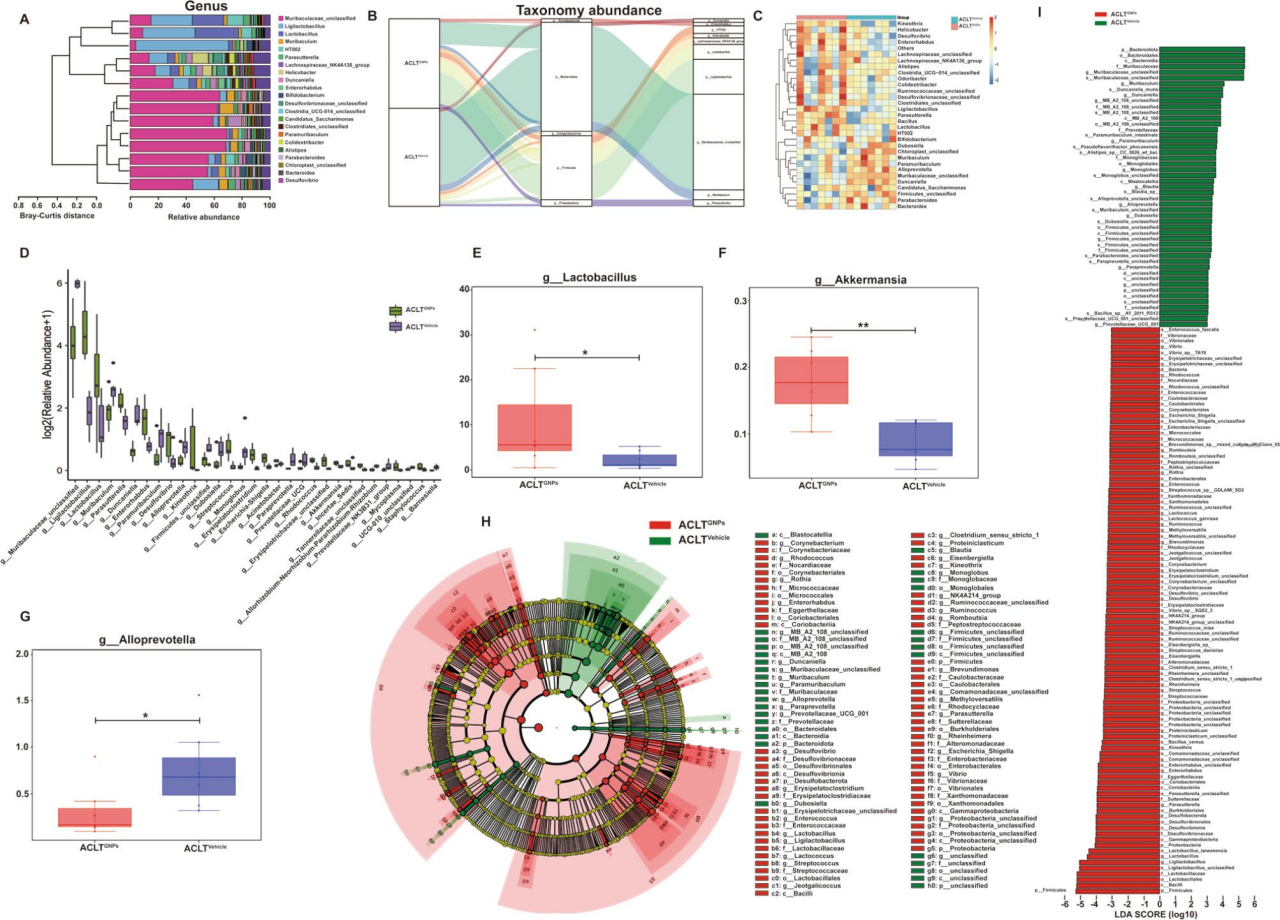
Fecal microbiota composition of ACLT^Vehicle^ and ACLT^GNPs^ mice at genus taxonomic level. (**A**) Relative abundance of gut microbiota at genus taxonomic level in ACLT^Vehicle^ and ACLT^GNPs^ groups. (**B**) Sankey plot of the taxonomic data changed with the breadth of the branch at genus (right side) and phylum (middle) levels in ACLT^Vehicle^ and ACLT^GNPs^ groups (left side). The color and width of the branches represents the flow of specific genera within different phyla. (**C**) Heatmap of different gut microbiota at genus taxonomic level between ACLT^Vehicle^ and ACLT^GNPs^ groups. Color in the heatmap is utilized to describe specific general abundance, with blue indicating lower abundance and red indicating higher abundance. (**D**) Statistical differences of gut microbiota at genus taxonomic level between ACLT^Vehicle^ and ACLT^GNPs^ groups were evaluated with box plots. (**E**) Relative abundance of genus *Lactobacillus* in each group was displayed by bar plots. (**F**) Relative abundance of genus *Akkermansia* in each group was displayed by bar plots. (**G**) Relative abundance of genus *Alloprevotella* in each group was displayed by bar plots. (**H**) A phylogenetic tree with cladogram computed by linear discriminant analysis effect size (LEfSe) algorithm depicted taxonomic association between microbiome communities from ACLT^Vehicle^ and ACLT^GNPs^ groups. The circles radiating from inside to outside represents the taxonomic level from Kingdom to Species. Each node on different levels represents the specific taxon, and the node diameter was proportional to the relative abundance. Yellow nodes represent no significant difference in species between the two groups. Red nodes indicate taxa predominant in ACLT^GNPs^ mice. Green nodes indica taxa predominant in ACLT^Vehicle^ mice. (**I**) LEfSe score indicated statistical differences in species between ACLT^Vehicle^ and ACLT^GNPs^ groups. (A-I) *n* = 7 samples per group. (*p < 0.05, **p < 0.01, ***p < 0.001, ****p < 0.0001, N.S represented not significant)

In addition, we aimed to perform a detailed analysis of the changes in gut microbiota at the genus level between the two groups ACLT^GNPs^ and ACLT^Vehicle^, and specifically identify the bacterial strains that are closely associated with the progression of OA. As is shown in Fig. 6A and C, the composition and structure of gut microbiota in ACLT^GNPs^ mice exhibited a completely different landscape at the genus level compared with ACLT^Vehicle^ mice. The Sankey plot depicted the gut microbiota composition between the two groups and showcased a distinct flow of microbial populations. We noticed that the dominant genera in ACLT^Vehicle^ mice included *Muribaculaceae_unclassified*, *Muribaculum*, *HT002*, and *Helicobacter*. However, in the ACLT^GNPs^ group, there is a pronounced shift, with the dominant genera *Lactobacillus*, *Ligilactobacillus*, and *Enterorhabdus* (Fig. 6B). The aforementioned alterations signified a significant remodeling in the gut microbiota composition, potentially related to GNPs-mediated OA-protective effects. We have identified and ranked the bacterial species in the gut microbiota of groups ACLT^GNPs^ and ACLT^Vehicle^ based on their varying abundances, which contributed to the observed variations in the gut microbiota landscape (Fig. 6D). Significantly, taxa such as *unclassified Muribaculaceae, Ligilactobacillus*, *Duncaniella*, *Parasutterella*, *Enterorhabdus*, *Muribaculum*, and *Lactobacillus* constitute over 1% of the total microbiota composition, suggesting their potential involvement in the pathogenesis of OA or in mediating the anti-osteoarthritic effects associated with GNPs. Additionally, we noticed that the genera *Lactobacillus* (2.15% vs. 10.64%, *p* = 0.035) and *Akkermansia* (0.09% vs. 0.18%, *p* = 0.006) occupied comparatively higher proportion in ACLT^GNPs^ group, while the genus of *Alloprevotella* (0.76% vs. 0.31%, *p* = 0.025) was enriched in ACLT^Vehicle^ group (Fig. 6E-G). Notably, the genus *Lactobacillus* has been widely proved to ameliorate OA and protect cartilage by modulating inflammation progression [64]. *Keun Hyung Cho* et al. demonstrated that *Lactobacillus* supplement increased the level of *Faecalibacterium* that produces butyrate, thereby improving OA progression [29]. The *InSug O Sullivan* group also revealed that *Lactobacillus* treatment could effectively alleviate OA-related joint pain, protect against cartilage degradation, and reverse gut microbiota dysbiosis [65]. *Rangru Liu et al.* found membrane vesicles from *Lactobacillus johnsonii* impede macrophage migration and M1 macrophage polarization in synovium to reduce inflammatory factor production, thereby mitigating inflammation, cartilage damage, and pain associated with OA [66]. As for *Akkermansia*, *Qi Wang et al.* found that it serves as a gut commensal probiotic bacterial species in oral chondroitin sulfate-mediated ameliorating effects on OA [67]. Interestingly, previous research proved that *Alloprevotella* is enriched in patients with Kashin-Beck disease, an endemic osteoarthritis in China. Here, *Alloprevotella* was markedly decreased by GNPs administration, which may be involved in the protective role of GNPs in OA [68]. 

To further illustrate the significant differences in gut microbiota composition between group ACLT^GNPs^ and ACLT^Vehicle^, and identify specific bacteria that played the dominant role in GNPs-induced anti-osteoarthritic effects, we performed linear discriminant analysis Effect Size (LEfSe) and Cladogram (based on maximum relative abundance difference in each level) analyses (Fig. 6H and I). Among the bacterial taxa that were enriched in ACLT^GNPs^ mice, we observed an overrepresentation of taxa such as the class of *Bacilli* (including the order of *Lactobacillales* and the family of *Enterococcaceae*, *Lactobacillaceae*, *Streptococcaceae*, and *Erysipelatoclostridiaceae*), *Desulfovibrionia* (from the order of *Desulfovibrionales* to the family of *Desulfovibrionaceae*), *Coriobacteriia* (including the order of *Coriobacteriales* and the family of *Eggerthellaceae*), and *Gammaproteobacteria* (including the order of *Enterobacterales*, *Burkholderiales*, *Vibrionales*, and *Xanthomonadales*, and the family of *Xanthomonadaceae*, *Vibrionaceae*, *Enterobacteriaceae*, *Alteromonadaceae*, *Sutterellaceae*, and *Rhodocyclaceae*), which may be linked to GNPs-mediated protective role against OA. Meanwhile, the ACLT^Vehicle^ group was characterized by an abundance of taxa such as the class of *Bacteroidia* (including the order of *Bacteroidales*, the family of *Prevotellaceae*) and MB_A2_108. These findings suggested that group ACLT^Vehicle^ and group ACLT^GNPs^ may have distinct microbiota profiles, which could contribute to differences in OA progression.

**Fig. 7:**
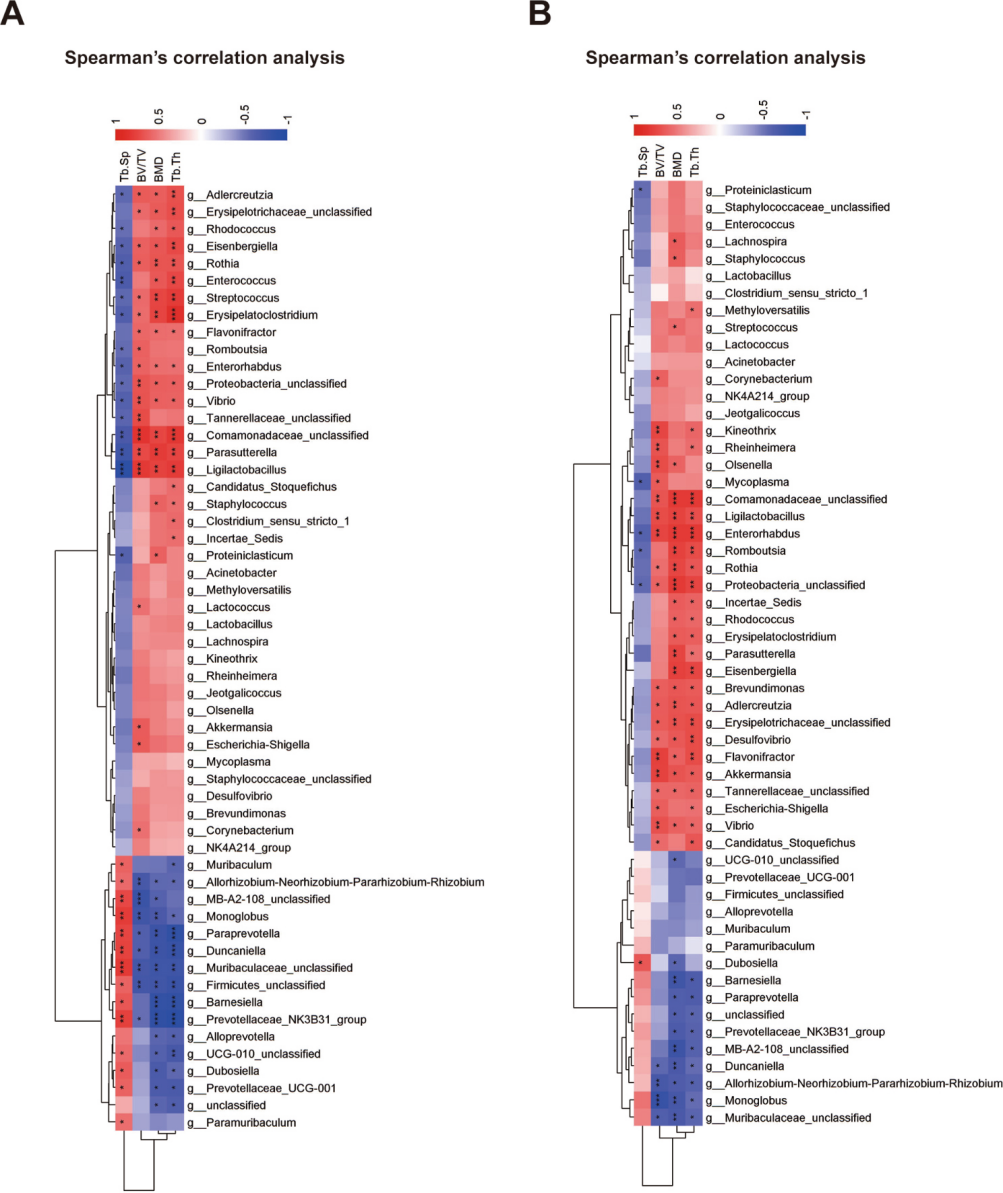
Spearman’s correlation analysis between gut microbiota and bone histomorphometry indexes. (**A**) Correlation analysis between gut microbiota at genus taxonomic level and femur-related indexes. Colorings represent the median Spearman correlation coefficient. (**B**) Correlation analysis between gut microbiota at genus taxonomic level and tibia-related indexes. Colorings represent the median Spearman correlation coefficient. (*p < 0.05, **p < 0.01, ***p < 0.001, ****p < 0.0001, N.S represented not significant)

Subsequently, we performed correlation analyses between individual microbial constituents and the integrity of subchondral bone encompassing both tibial and femoral sites, which were rendered as high-definition heatmaps, detailly illustrating the strength and directionality of associations. In the heatmap dedicated to the femoral findings, the genera of *Adlercreutzia*, *Eisenbergiella*, *Rothia*, *Streptococcus*, *Erysipelatoclostridium*, *Parasutterella*, and *Ligilactobacillus* manifested a salient and positive correlation with the BMD measurements, indicating a plausible protective or compensatory interaction with femoral bone sustenance. Conversely, the genera of *Monoglobus*, *Paraprevotella*, *Duncaniella*, *Barnesiella*, *Prevotellaceae_NK3B31_group*, and *Alloprevotella* exhibited a robust and positive correlation, implying a potential contributory role in the pathophysiology of femoral subchondral bone compromise (Fig. 7A). Within the tibial landscape, the genera of *Ligilactobacillus*, *Enterorhabdus*, *Rothia*, *Brevundimonas*, *Adlercreutzia*, *Desulfovibrio*, *Flavonifractor*, *Akkermansia* and *Vibrio*, emerged as an ostensibly beneficial presence, with significant positive correlations underscoring its alignment with preserved or enhanced tibial bone architecture. Adjacently, the genera of *Monoglobus*, *Duncaniella*, and *Barnesiella*, as a putative deleterious agent, showcased a consistent and inverse relationship with the quantified indices of BMD and Tb.Th (Fig. 7B). These findings not only contributed to the burgeoning narrative connecting the gut microbiota to systemic skeletal phenomena but also paved avenues for therapeutic interrogations targeting microbiota as potential modulators in the alleviation or reversal of OA-related subchondral bone loss.

### GNPs treatment increased the abundance of metabolite SCFAs

**Fig. 8:**
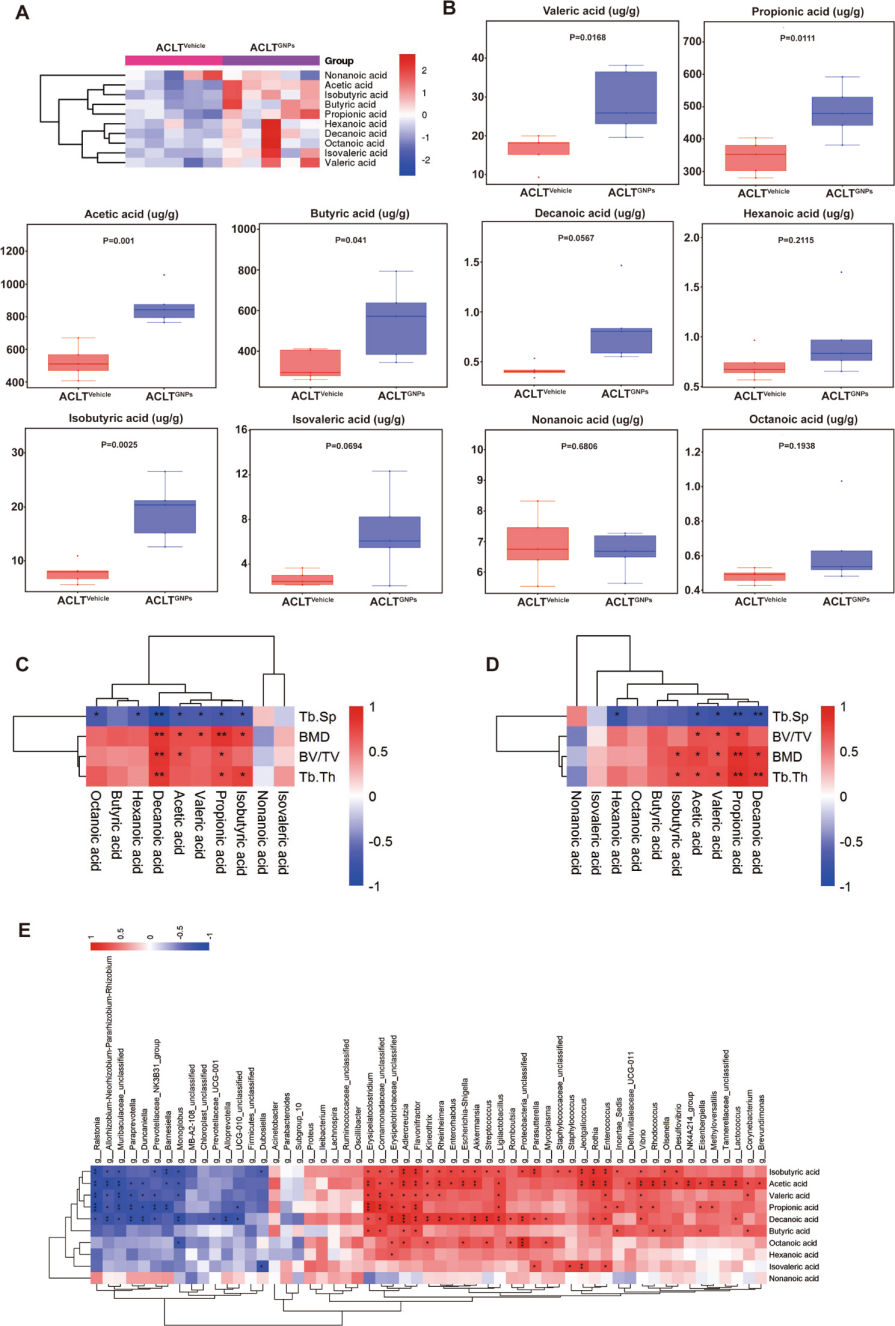
GNPs treatment changed the profile of SCFA metabolism in ACLT-induced OA mice. (**A**) Heatmap of SCFAs between ACLT^Vehicle^ and ACLT^GNPs^ groups. The blue indicates lower abundance and red indicates higher abundance. (**B**) The concentration of SCFAs including valeric acid, propionic acid, acetic acid, butyric acid, decanoic acid, hexanoic acid, isobutyric acid, isovaleric acid, nonanoic acid, and octanoic acid from feces between ACLT^Vehicle^ and ACLT^GNPs^ mice. (**C**) Correlation analysis between SCFAs and femur-related indexes. Colorings represent the median Spearman correlation coefficient. (**D**) Correlation analysis between SCFAs and tibia-related indexes. Colorings represent the median Spearman correlation coefficient. (**E**) Correlation analysis between SCFAs and gut microbiota at genus taxonomic level. Colorings represent the median Spearman correlation coefficient. *n* = 7 samples per group. (*p < 0.05, **p < 0.01, ***p < 0.001, ****p < 0.0001, N.S represented not significant)

Previous studies demonstrated that the gut microbiota dominating in ACLT^GNPs^ group such as *Lactobacillus*, *Akkermansia*, and *Muribaculum* were involved in the production of SCFAs [69–72]. In consideration of the key role of SCFAs in maintaining the homeostatic balance of intestine flora and the beneficial effects of SCFAs on alleviating OA progression, we investigated the alteration in the SCFAs concentration in fecal samples via a targeted metabolomics assay [29, 73]. Consistent with the alteration in gut microbiota structure and composition, the PCA score plot and Orthogonal partial least-squares discriminant analysis (OPLS-DA) score plot demonstrated distinct clustering patterns, indicating a pronounced dissimilarity in the SCFA metabolism between the two groups (Fig. S5A-5B). Among these SCFAs, valeric acid (*p* = 0.0168), propionic acid (*p* = 0.0111), acetic acid (*p* = 0.001), butyric acid (*p* = 0.041), and isobutyric acid (*p* = 0.0025) manifested higher amounts in the ACLT^GNPs^ group. Although other SCFAs including decanoic acid (*p* = 0.0567), hexanoic acid (*p* = 0.2115), isovaleric acid (*p* = 0.0694) and octanoic acid (*p* = 0.1938) exhibited no marked differences between the two groups, they showed a higher trend of abundance in the ACLT^GNPs^ group. Therefore, compared to the ACLT^Vehicle^ group, there was a significant increase in the total metabolites of SCFAs in the feces of the ACLT^GNPs^ group (Fig. 8A and B and S5C). Our correlation analysis of SCFAs within the gut revealed significant interrelationships, indicative of complex microbial interactions. A notable positive correlation between butyric acid and propionice acid, acetic acid, as well as isobutyric acid suggested a linked metabolic pathway or mutualistic microbial activities. In contrast, an inverse relationship between propionic acid and butyric acid may reflect competitive dynamics for substrates within the microbial community. These findings, visualized in a correlation matrix, underscored the importance of SCFAs in gut ecology and the potential of modulating SCFA profiles to influence health outcomes (Fig. S5D).

To determine the implications of SCFAs on OA progression, we conducted a correlation analysis between these SCFAs and the histomorphometry indexes for subchondral bone in both tibia and femur. Notably, we observed that specific SCFAs such as decanoic acid, propionic acid, valeric acid, and acetic acid displayed a robust positive association with the BMD of subchondral bone in the tibia and femur, indicating a potential role for these SCFAs in maintaining the integrity of subchondral bone (Fig. 8C and D). These findings not only underscored the potential of SCFAs as biomarkers or modulators in OA, but also enriched our understanding of the metabolic pathways influencing bone architecture.

Subsequently, we performed an integrated microbiome-metabolome analysis to elucidate complex interplays between distinct microbial genera and multiple SCFAs. As shown in Fig. 8E, 46 genera displayed significant correlations with SCFA abundance. Among these genera, we observed that 34 genera were positively associated with multiple SCFAs while 12 genera showed strong negative correlations with SCFAs. Combining the bacteria abundance (> 0.5%) and differential distribution (*p* < 0.05), the genera of *Ligilactobacillus*, *Parasutterella*, *Enterorhabdus*, and *Desulfovibrio* were marked as positively correlated with the level of SCFAs, while *Muribaculaceae_unclassified*, *Duncaniella*, and *Alloprevotella* exhibited negative associations. Consistent with previous research, we observed that *Desulfovibrio*, a potent generator of acetic acid, showed a strong positive correlation with acetic acid and isobutyric acid levels [74]. Additionally, *Ligilactobacillus* as a key participant in the production of SCFAs, were substantially positively linked to acetic acid, valeric acid, propionic acid, decanoic acid, and isobutyric acid [75, 76]. Indeed, other genera such as *Akkermansia*, *Flavonifractor*, *Adlercreitzia*, and *Kineothrix* were also widely positively associated with multiple SCFAs, but their regulatory effects on SCFA metabolism may be little due to their low abundance (mean abundance < 0.5%). Collectively, these results illuminated the multifaceted relationships between the gut microbiome and SCFA production, suggesting that the modulation of the gut microbiota could be a promising avenue for influencing SCFA profiles and bone health.

### GNPs maintained M1/M2 macrophage balance, restrained the release of proinflammatory cytokines, and improved the intestinal barrier function

**Fig. 9:**
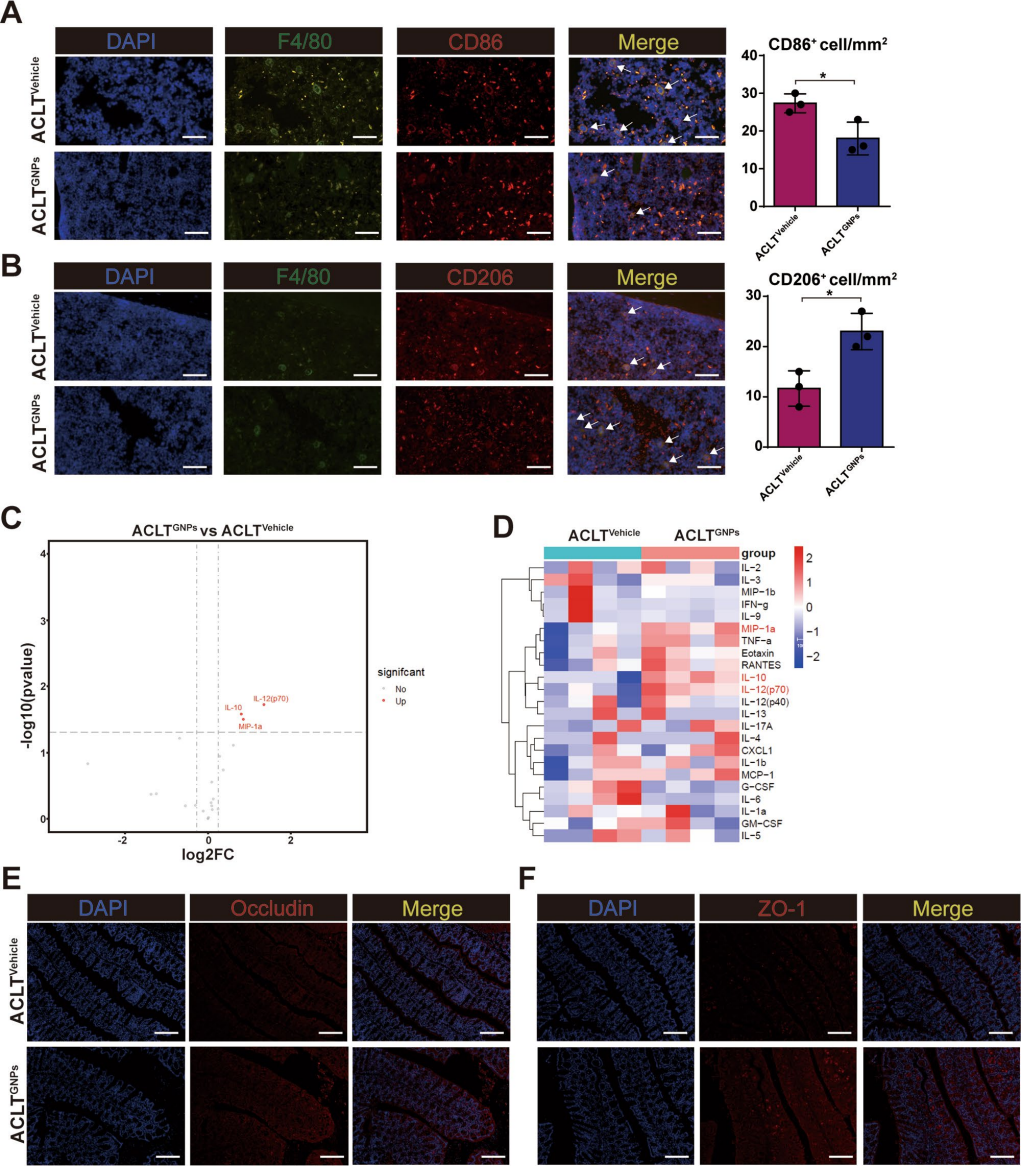
GNPs reversed the balance of M1/M2 macrophage polarization and improved gut-barrier function. (**A**) CD86 (M1 macrophage marker) distribution in subchondral bone were detected by immunofluorescence in the two groups. (**B**) CD206 (M2 macrophage marker) distribution in subchondral bone were detected by immunofluorescence in the two groups. (**C**) Volcano plot of cytokine alteration at serum was measured by mouse inflammation array. (**D**) Heatmap representation of cytokine levels from ACLT^Vehicle^ and ACLT^GNPs^ mice. (**E**) Representative immunofluorescence images showing in situ expression of Occludin. (**F**) Representative immunofluorescence images showing in situ expression of ZO-1. (*p < 0.05, **p < 0.01, ***p < 0.001, ****p < 0.0001, N.S represented not significant)

Macrophages are a type of white cells responsible for the engulfment and digestion of pathogens, cellular debris, and cancerous cells, which play a key role in inflammation diseases [77]. Macrophages are highly plastic and could adopt different functional states in different microenvironment, commonly referred to as polarization. Generally, the two main subpopulations of polarized macrophages consist of classically activated (M1) macrophages and alternatively activated (M2) macrophages according to the environment, transcription factors, and cytokines secreted by macrophages [78]. M1 macrophages are characterized by pro-inflammatory cytokine production and functions in host defense and antitumor immunity. In contrast, M2 macrophages, recognized for their expression of scavenger receptors and anti-inflammatory factors, facilitate tissue remodeling and support immunosuppressive micro-environment [79, 80]. Recent studies demonstrated that M1 macrophages amassed within the dorsal root ganglia (DRG), hosting the neuronal cell bodies linked to the sensory network of the injured OA knee joint, and this infiltration instigated ongoing pain promotion and contributed to the OA progression [81]. Additionally, apoptotic bodies originating from M2 macrophages have the capacity to safeguard joint cartilage from deterioration and ameliorate OA progression by counteracting the inflammatory response elicited by M1 macrophages [82]. These findings have proved that the M1-to-M2 macrophage phenotype transition may perform an important role in attenuating bone and cartilage deconstruction in OA [83, 84]. Given that SCFAs contribute to a remarkable immunomodulatory effect by inducing suppression of the M1 macrophage phenotype and facilitating the polarization towards the M2 phenotype, we have examined the alterations in the M1/M2 macrophage population within the femoral bone marrow of two groups of mice by employing immunofluorescence staining techniques [85]. Our observations have indicated a significant diminution in the M1 macrophage subset, marked by co-expression of F4/80 and CD86, in the ACLT^GNPs^ group when juxtaposed with the ACLT^Vehicle^ cohort. Conversely, there was a concomitant increase in the M2 macrophage subset, identified by the presence of F4/80 and CD206 markers, in the ACLT^GNPs^ group relative to the ACLT^Vehicle^ group. These findings suggested that GNP intervention may exert modulatory effects on the macrophage polarization within the femoral bone marrow, potentially implicating a shift towards an anti-inflammatory M2 phenotype conducive to tissue repair and regeneration processes (Fig. 9A and B).

Subsequently, we detected the profile of inflammatory cytokines and chemokines in the serum sample via cytokines array, thereby revealing differential expression patterns of pro-inflammatory and anti-inflammatory mediators between the two groups. Among these cytokines, the concentrations of macrophage inflammatory protein-1 alpha (MIP-1α), IL-12(p70) and IL-10 were significantly elevated in the ACLT^GNPs^ group. Notably, IL-10 is an anti-inflammatory cytokine known for its critical role in suppressing pro-inflammatory responses and facilitating the M2 macrophage polarization. This upregulated expression of IL-10 coincided with the augmented presence of M2 macrophages, as previously noted in the same experimental group (Fig. 9C) [86]. Additionally, we observed that proinflammatory cytokines such as granulocyte colony-stimulating factor (G-CSF) and IL-6 were decreased with GNPs administration, which plays a crucial role in M1 macrophage polarization (Fig. 9D) [87]. These results further reinforced the macrophage polarization observations and collectively suggested an immunomodulatory effect induced by the treatment in the ACLT^GNPs^ group, moving towards an environment that may improve inflammation and OA progression.

Generally, an imbalance within the gut microbiota fosters a heightened porousness in the intestinal epithelium, enhances intestinal permeability, and thereby facilitates the seepage of pernicious microbes and metabolites into circulation [88, 89]. *Boer C.G.et al*. demonstrated that enhanced permeability of the intestinal barrier permits the entry of microbial by-products that activate macrophages and intensify inflammation within the joints, thereby aggravating OA [13]. In addition, previous research has proved that SCFAs provide essential energy sources for colonic epithelial cells and orchestrate a symphony of molecular signals, which enhance the fortitude of the intestinal barrier [90, 91]. In order to elucidate the impact of GNPs administration on intestinal permeability, we investigated the expression of zonula occludens-1 (ZO-1) and occludin in colonic tissue via immunofluorescence. Compared with ACLT^Vehicle^ mice, GNPs substantially upregulated the expression of these critical tight junction proteins, which denoted a notable enhancement in gut barrier functionality (Fig. 9E and F). Overall, GNPs treatment improved OA progression by regulating macrophage polarization, the production of cytokines, and intestinal-barrier function, which may be linked to the increased SCFA levels.

## Discussion

As is known to all, the destruction of gut microbiota homeostasis is often a common underlying characteristic of numerous diseases [92, 93]. With continuous development and refinement of the pathological basis in OA, growing evidence suggests that OA is closely associated with gut microbiota imbalance, metabolic disorders, and intestinal barrier damage [94, 95]. As one of the important metabolites of gut microbiota, SCFAs can enter joint tissues through blood circulation, and have been demonstrated to be widely involved in OA progression [96]. Herein, our study demonstrated a close relationship among GNPs, gut microbiota, and bone metabolism, supporting that GNPs could noticeably alleviate the severity of OA induced by ACLT in mouse models through a “microbiota-gut-joint” axis dependent manner. The intervention of GNPs could change the abundance and diversity of OA-associated gut microbiota, increase the content of gut microbiota-related metabolites SCFAs (such as acetic acid, propionic acid, butyric acid, isobutyric acid, and valeric acid), improve intestinal permeability, and modulate the dynamic balance of M1 and M2 macrophages, thus further reducing the level of proinflammatory cytokines in the joint, and eventually ameliorating the severity of OA. In addition, the elimination of gut microbiota by antibiotics mixture administration and the alteration of microflora composition by FMT further confirmed that the above process is carried out by a gut microbiota-dependent mechanism. Therefore, our findings supported the feasibility of using GNPs as a gut microbiome modulator to safely and effectively improve the severity of OA.

Recent animal studies have demonstrated that dysregulation of gut microbiota can affect bone metabolism as well as promote the development of OA after joint injury [97]. And consistent associations were observed between alterations in *Ruminococcaceae*, *Faecalibacterium*, and *Fusobacterium* with both inflammatory biomarkers and histological OA severity in mice [14]. In addition, human studies have also proved that gut microbiota dysbiosis is significantly associated with OA and OA-related symptoms at different joint sites [11, 13, 98]. Therefore, we used ACLT mice as an ideal OA model to investigate therapeutic strategies targeting gut microbiota disturbance. As a kind of nanomaterial with illustrious biocompatibility [99], GNPs play a dramatic role in microflora regulation [100–102], but there are few studies about their gut microbial modulation in OA. In this study, we found that GNP intervention significantly altered the diversity and species richness of gut microbiota in mice, manifested by an increase in the abundance of *Firmicutes*, *Verrucomicrobiota*, and *Proteobacteria*, which is consistent with previous research findings [103], and may be beneficial to modulate gut microbiota dysbiosis [104]. At the genus taxonomic level, GNPs increased the relative abundances of *Acinetobacter*, *Akkermansia*, *Desulfovibrio*, *Ligilactobacillus*, *Lactobacillus*, and *Parasutterella*, while decreased *Alloprevotella*, *Duncaniella*, and *Paraprevotella* abundances. These bacteria exert different effects on gut metabolism and disease development, and are widely involved in the regulation of gut barrier and intestinal inflammation, metabolic disorders, neurodegenerative diseases, and cancers [105–108]. Of note, we found a significant positive correlation between the abundance of *Ligilactobacillus* and the improvement of both femur-related indexes and tibia-related indexes among these bacteria. And *Ligilactobacillus* has been identified to be able to safeguard gut barrier [109], as well as diminish serum levels of inflammatory cytokines and bacterial translocations [110, 111]. Moreover, to investigate whether the anti-osteoarthritic effects exerted by GNPs depend on gut microbiota, we first used ABX to remove gut microbiota, and found that the anti-osteoarthritic effects of GNPs were successfully blocked. Next, gut microbiota-depleted mice were transplanted with the intestinal flora from ACLT^Vehicle^ and ACLT^GNPs^ mice. As we expected, FMT reproduced the mitigation effect of GNPs on OA. Therefore, the above results demonstrated that gut microbiota indeed contributed to the anti-osteoarthritic effects of GNPs on OA.

SCFAs are the main metabolites derived from microbial fermentation of dietary fibers in the intestine, closely related to changes in gut microbiota, and possess immunomodulatory capabilities [112]. Additionally, it is known that immune activation is intimately related to bone homeostasis [113]. Based on these, *Lucas, S* et al. believed that SCFAs acted as effective regulators of OC metabolism and bone homeostasis, which could alleviate the progression of OA and prevent aberrant inflammatory bone loss [26]. Mechanistically, SCFAs (such as propionate and butyrate) induce metabolic reprogramming of OCs, leading to enhanced glycolysis at the expense of oxidative phosphorylation, thus downregulating essential OC genes (including TRAF6 and NFATc1), ultimately providing a direct mechanistic link between the gut microbiota and bone [26]. In this study, SCFAs-targeted metabolomics analysis suggested that GNP intervention significantly increased the production of gut microbiota-derived SCFAs, which were consistent with the alteration in gut microbiota. Interestingly, we observed that *Ligilactobacillus* showed a clear correlation with most types of SCFAs. As a class of gram-positive bacilli, *Ligilactobacillus* is a well-characterized bacteriocin producer and probiotic organism [114]. It is reported that *Ligilactobacillus* abundance was positively related to SCFAs, especially acetic acid, propionic acid, and butyric acid contents [115]. In addition, it is generally believed that overexpression of MMPs induced by pro-inflammatory cytokines such as IL-1β is closely related to the destruction of articular cartilage matrix in chondrocytes from patients with OA [116]. Based on the study of *W. Bo et al.*, sodium butyrate can inhibit the up-regulation of MMP-1, MMP-3, and MMP-13 induced by IL-1β at both the gene and protein levels, thus alleviating the degradation of type II collagen in human chondrocytes [28]. Mechanistically, sodium butyrate impeded the activation of NF-κB by suppressing the phosphorylation of IKK, IκBα, and NF-κB p65, thereby affecting the pathological development of OA. The above results further confirmed the significant role of the gut microbiota-metabolites axis in the anti-osteoarthritic effects exerted by GNPs.

Moreover, we also found that GNP intervention conspicuously increased the number of M2 macrophages while decreased M1 macrophages, thereby lowering the ratio of M1/M2, which aligned with the changes in metabolism of SCFAs. Recent studies have identified that SCFAs exert important effects on macrophage polarization, manifested by propionic acid, butyric acid, and valeric acid inhibiting M1 macrophage polarization and promoting M2 macrophage polarization, thus exhibiting anti-inflammatory effects [85, 117, 118]. In this study, the reduced M1/M2 ratio may be induced by the high levels of SCFAs. Modulating the polarization of macrophages can reduce synovial inflammation, improve joint pain, as well as restrain articular cartilage degeneration, thus alleviating the progression of OA [119]. Additionally, we analyzed cytokine microarray assay and found that GNPs significantly upregulated the level of anti-inflammatory cytokines IL-10, which corresponded to the polarization of macrophages and alterations in SCFAs. And the up-regulation of SCFAs may be the reason for the increase in anti-inflammatory cytokines [90]. However, the specific mechanisms of GNPs affecting the crosstalk of SCFAs and anti-inflammatory cytokines need to be further explored.

**Fig. 10:**
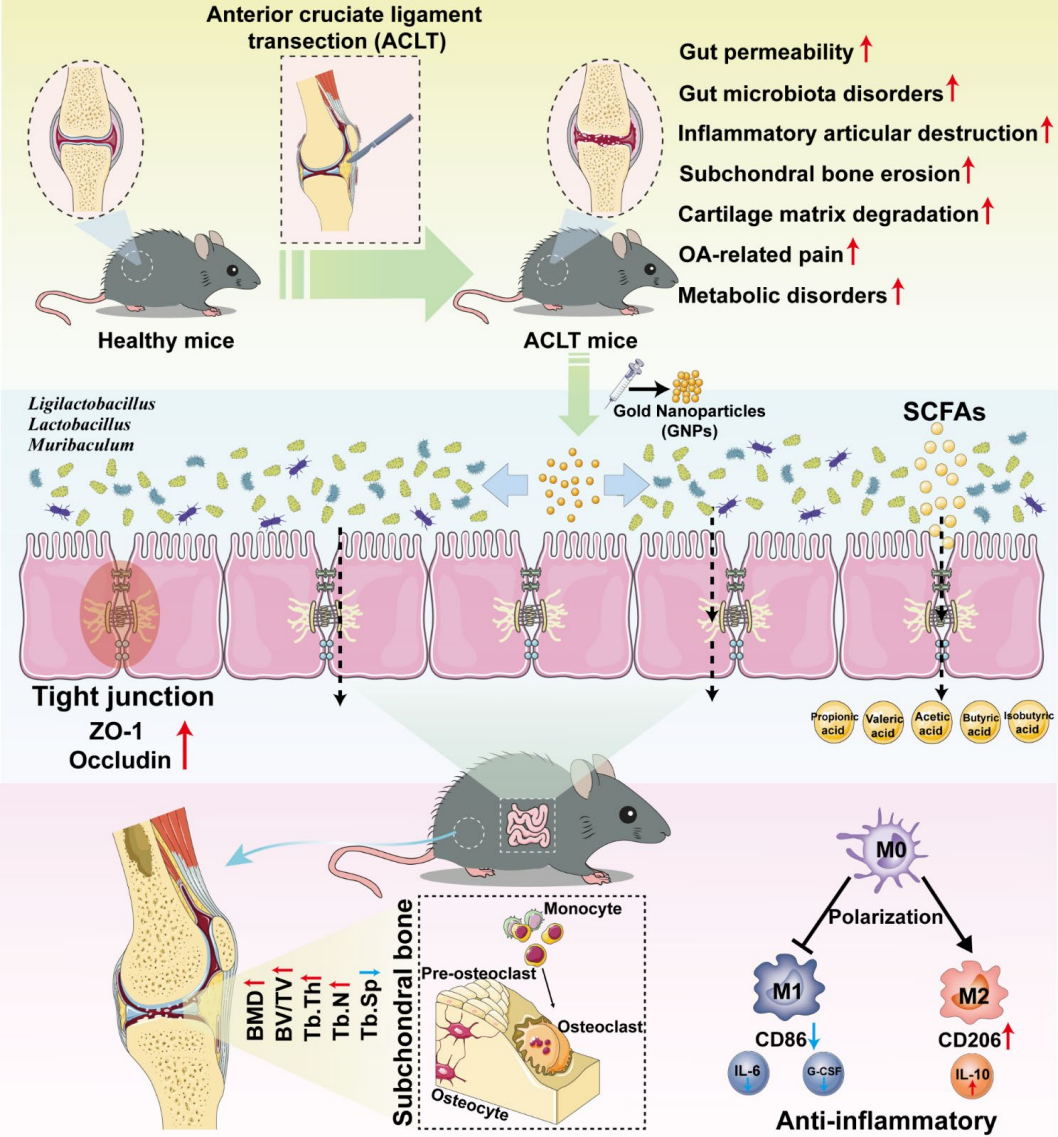
Schematic for GNPs-mediated protective effects on OA progression. GNPs treatment reshaped the landscape of gut microbiota, increased SCFA production and the release of anti-inflammatory cytokines in serum, improving M1/M2 macrophage balance and restoring gut barrier function

In fact, there are some limitations in this study: (i) We used feces samples as representative samples, but this may not fully reflect the distribution of gut microbiota in different regions of the intestine. (ii) Although the use of ABX has been shown to have lasting effects on gut microbiota and deplete almost all bacteria, it does not necessarily meet the requirements of germ-free [120]. (iii) We selected the vital gut microbiota (such as *Ligilactobacillus*), which play an essential role in the anti-osteoarthritic effects of GNPs, however, the specific mechanism by which the gut microbiota itself or extracellular vesicles regulate the progression of OA remains unclear. (iv) Other types of MNPs can be used for performance comparison with GNPs, which contributes to verifying the good anti-osteoarthritic effects of GNPs. (v) As mentioned above, we analyzed the interaction between gut microbiota along with its metabolites (SCFAs) and macrophage polarization after GNPs intervention. The precise impact of GNPs-mediated metabolic alternations on osteoclastogenesis remains elusive, and it may offer valuable perspectives for future investigations on this topic. In summary, administration with GNPs could alleviate ACLT-induced OA by reshaping gut microbiota and improving intestinal permeability (Fig. 10). The underlying protective mechanism was related to the macrophage polarization and increased levels of anti-inflammatory cytokines mediated by the increased SCFA content. Our data confirmed that GNPs served as an effective regulator of gut microbiota, which induced alterations in the abundance of key microorganisms in the gut, closely related to SCFA metabolism, thus endowing GNPs with a novel mechanism to reduce inflammatory response and mitigate OA progression. The treatment of OA is a global clinical problem, and there is no recognized optimal disease-modifying therapy. Although the specific mechanism by which GNPs modulate gut microbiota needs to be further investigated, a series of therapy strategies derived from GNPs still provide a potentially novel candidate approach and lay an important theoretical foundation for future OA solutions. Furthermore, utilizing GNPs as innovative modulators of the gut microbiome could redefine treatment strategies for OA by leveraging the “microbiota-gut-joint” axis, offering a promising alternative to conventional therapies focused mainly on symptom relief and joint preservation. It is necessary to optimize GNPs formulations for safety and efficacy in humans, establish appropriate dosing guidelines that account for the variability in human gut microbiota, and develop reliable, non-invasive methods to monitor the alterations in gut microbiota and the progression of OA in response to GNPs treatment. Additionally, the exploration of GNPs interactions with specific components of the gut microbiota, and how these interactions influence systemic inflammation and OA progression is crucial, which help improve GNPs formulations for enhanced specificity and reduced side effects. The construction of GNPs-based modulation of the gut microbiome as a treatment for OA is complex and challenging, which may be a significant stride towards innovative therapeutic modalities for OA.

### Electronic supplementary material

Below is the link to the electronic supplementary material.

Supplementary Material 1

Supplementary Material 2

Supplementary Material 3

Supplementary Material 4

Supplementary Material 5

## Data Availability

The data that support the findings of this study are available within the article and its supplementary materials. Raw data are available from the corresponding authors upon reasonable request.

## Abbreviations

ABX: Antibiotics
ACLT: Alleviate anterior cruciate ligament transection
AD: Alzheimer’s disease
BMD: Bone mineral density
BSE: backscattered electron
BV/TV: Bone volume fraction
COX-2: Cyclooxygenase-2
CGRP: Calcitonin gene-related peptide
COG: Clusters of orthologous groups
DRG: Dorsal root ganglia
EDTA: Ethylenediaminetetraacetic acid
FMT: Fecal microbiota transplantation
G-CSF: Granulocyte colony-stimulating factor
GNPs: Gold nanoparticles
H&E: Hematoxylin and eosin
IL: Interleukin
IHC: Immunohistochemistry
LA-1: Lactobacillus
LEfSe: Linear discriminant analysis effect size
LIF: Leukemia inhibitory factor
MIP-1α: Macrophage inflammatory protein-1 alpha
MMP-13: Metalloproteinase-13
MNPs: Metallic nanoparticles
NSAIDs: Nonsteroidal anti-inflammatory drugs
OA: Osteoarthritis
OARSI: Osteoarthritis research society international
OCs: Osteoclasts
OP: Osteoporosis
OPLS-DA: Orthogonal projections to latent structures discriminate analysis
OTUs: Observed taxonomic units
PCA: Principle component analysis
PCoA: Principal coordinate analysis
PGF: Pseudo-germ-free
PICRUSt2: Phylogenetic investigation of communities by reconstruction of unobobserved states 2
RA: Rheumatoid arthritis
ROI: Region of interest
SNPs: Silver nanoparticles
SPF: Specific pathogen-free
Tb.Th: Trabecular thickness
Tb.Sp: Trabecular separation
TiO_2_: Titanium dioxide
TNF: Tumor necrosis factor
TRAP: Tartrate-resistant acid phosphatase
ZnO: Zinc oxide
ZO-1: Zonula occludens-1
