# Supplementary figures and images for: Gold nanoparticles exhibit anti-osteoarthritic effects via modulating interaction of the “microbiota-gut-joint” axis
Source: J Nanobiotechnology. 2024 Apr 8;22:157. doi: 10.1186/s12951-024-02447-y (PMC11000357; doi:10.1186/s12951-024-02447-y)

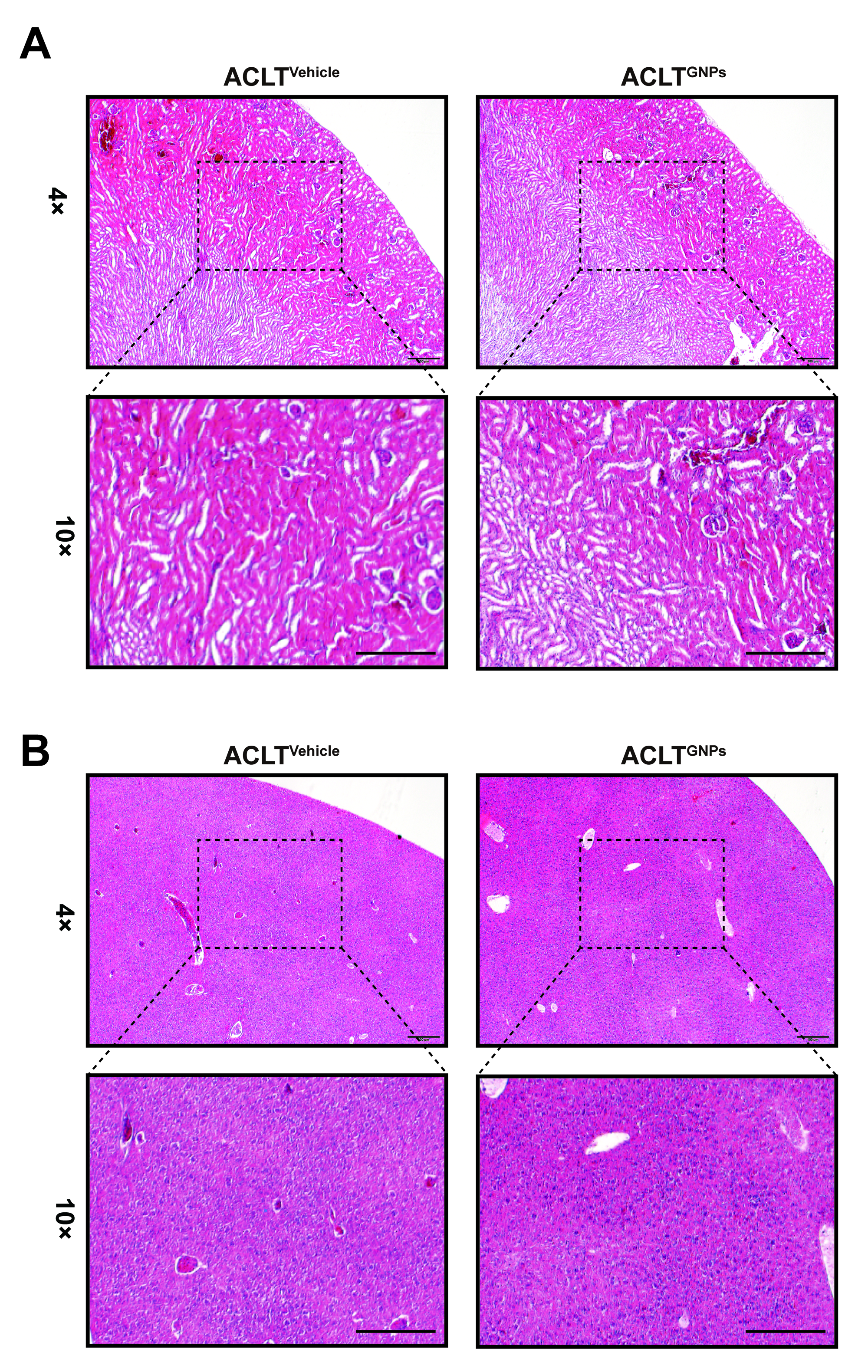

Supplement: Supplementary file 1 — Supplementary Material 1 [file 12951_2024_2447_MOESM1_ESM.tif]

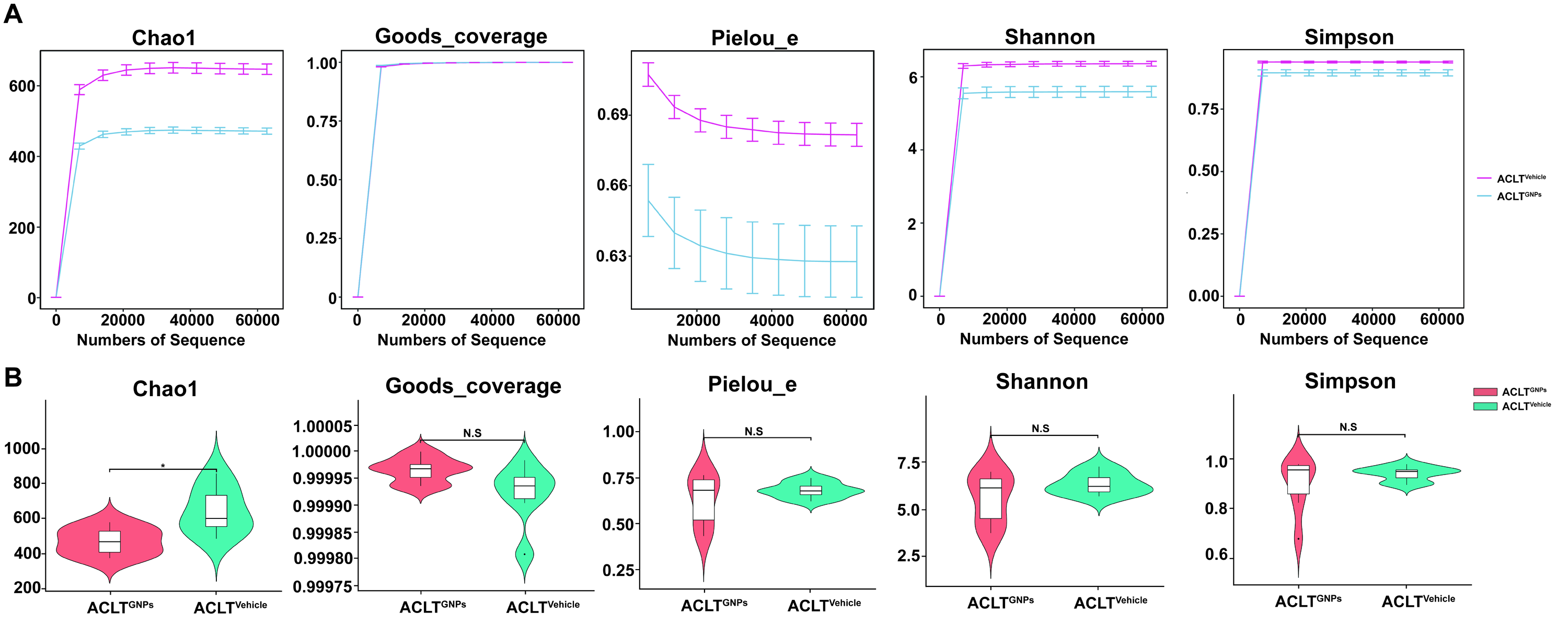

Supplement: Supplementary file 2 — Supplementary Material 2 [file 12951_2024_2447_MOESM2_ESM.tif]

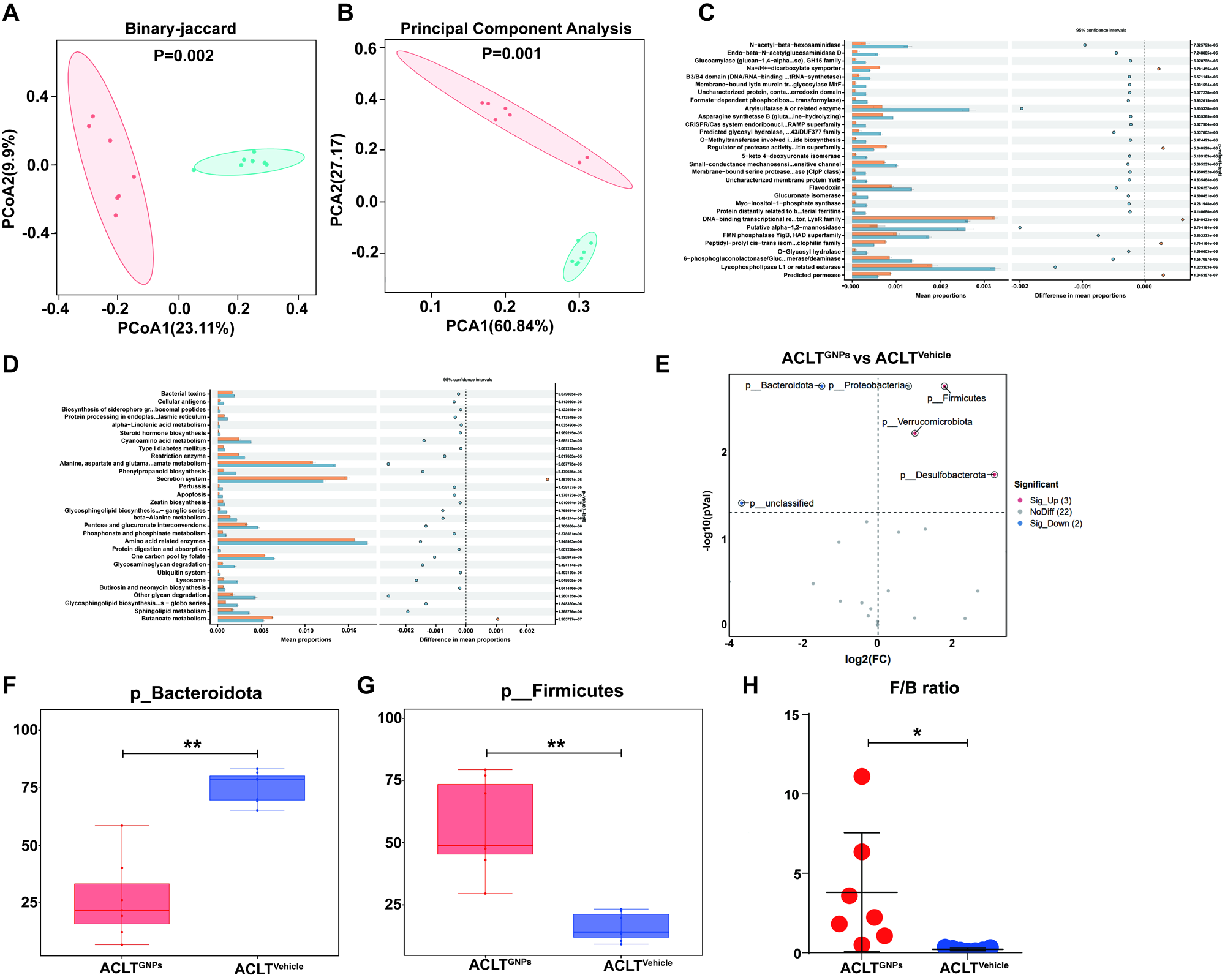

Supplement: Supplementary file 3 — Supplementary Material 3 [file 12951_2024_2447_MOESM3_ESM.tif]

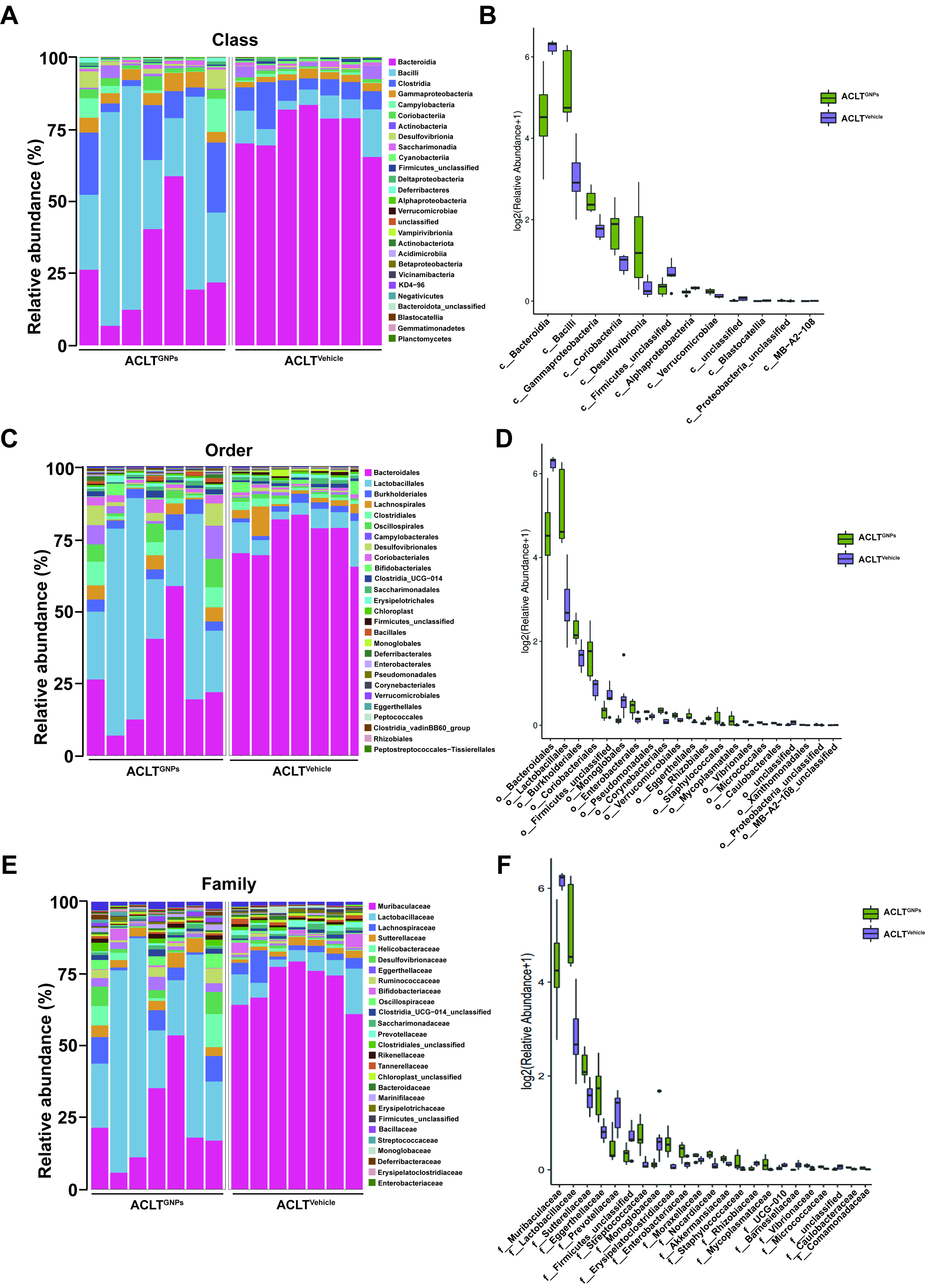

Supplement: Supplementary file 4 — Supplementary Material 4 [file 12951_2024_2447_MOESM4_ESM.tif]

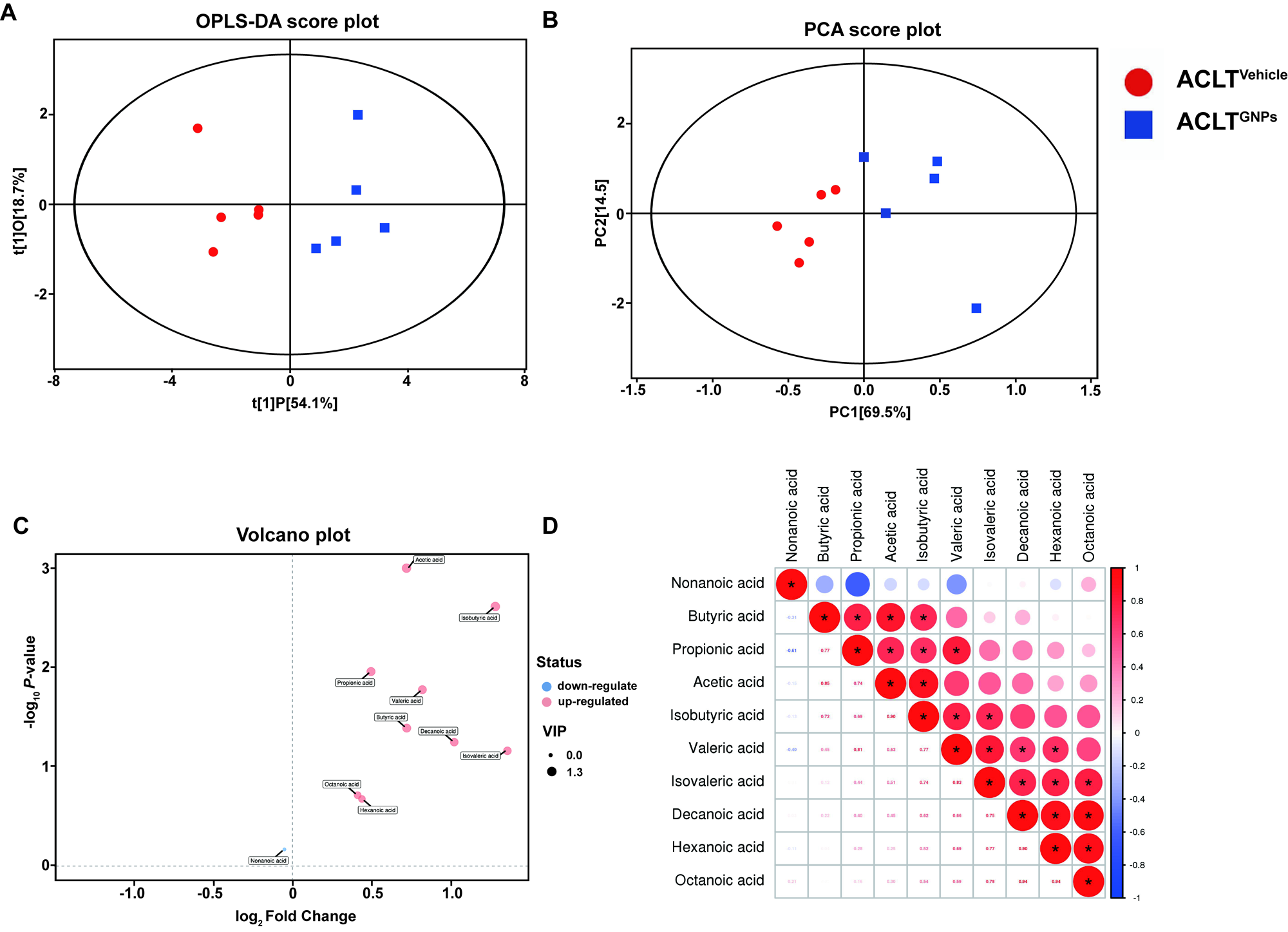

Supplement: Supplementary file 5 — Supplementary Material 5 [file 12951_2024_2447_MOESM5_ESM.tif]
